# Provably Secure Identity-Based Multi-Proxy Signature Scheme in the Standard Model for UAV-Assisted Rescue

**DOI:** 10.3390/s26216847

**Published:** 2026-10-29

**Authors:** Weifeng Long, Yunyun Qu, Yaying Wu, Ziran Yan, Na Wang

**Affiliations:** 1 School of Mathematical Sciences, Guizhou Normal University, Gui’an New District, Guiyang 550025, China; 460143769@gznu.edu.cn (W.L.); 243100060037@gznu.edu.cn (Y.W.); yzr@gznu.edu.cn (Z.Y.); 2 Guizhou Key Laboratory of NewGen Cyberspace Security, Guiyang 550001, China; 3 School of Cyber Science and Technology, Beihang University, Beijing 100191, China

**Keywords:** identity-based multi-proxy signature, provable security, standard model, UAV-assisted rescue

## Abstract

Identity-based multi-proxy signatures (IBMPS) enable an original signer to delegate signing authority to multiple proxy signers, who jointly sign a common message on the original signer’s behalf using their identity-associated private keys. This supports authenticated collaborative reporting in unmanned aerial vehicle (UAV)-assisted rescue. We propose an IBMPS scheme for this application and a security model that captures adaptive queries and signer collusion. Under the computational Diffie–Hellman (CDH) assumption and the collision resistance of ${\hat{H}}_{2}$ and ${\hat{H}}_{3}$, we prove unforgeability for the three forgery scenarios of Game 1 in the standard model (SM), with explicit reduction bounds and no public-key replacement. We also analyze long-term private-key recovery under proxy signing-key exposure, assuming an honestly generated target public key that remains unchanged. The scheme verifies individual partial signatures before aggregation, while final verification requires a constant number of pairings and scalar multiplications; signer-list processing still depends on group size. Under historical benchmark timings and the adopted operation counts, with ten proxy signers, the estimated total computational cost is 19.34–44.00% lower than that of five of the six baseline schemes, and the estimated final verification cost is 50.26–74.06% lower than that of all six. Under the adopted encoding assumptions, the 20-byte long-term private-key representation is the smallest among the compared schemes, the 276-byte per-proxy delegation ties for the smallest, and the 532-byte final signature matches that of Shin et al.’s scheme. The cost figures are model-based estimates, and the byte counts are per-object representation sizes rather than complete end-to-end communication costs.

## 1. Introduction

Natural disasters continue to cause substantial loss of life and extensive property damage worldwide. The severe floods in Central Africa and wildfires in the Amazon rainforest in 2019, together with the extreme heatwaves in North America in 2021, underscore the importance of timely disaster assessment and effective emergency response. Unmanned aerial vehicles (UAVs) are increasingly deployed to collect information from disaster-affected areas and support search-and-rescue operations. Depending on their platforms and payloads, UAVs offer advantages such as rapid deployment, wide-area coverage, nighttime operation, precise delivery of emergency supplies, and relatively low operating costs. These capabilities make UAVs valuable tools for disaster assessment and rescue coordination [1,2,3,4].

In UAV-assisted rescue missions, the ground control center relies on timely and authenticated reports about disaster locations and people in distress to inform operational decisions. An identity-based multi-proxy signature (IBMPS) scheme supports this requirement by allowing a designated group of UAVs to jointly sign a common report under signing authority delegated by a rescue commander.

**Figure 1:**
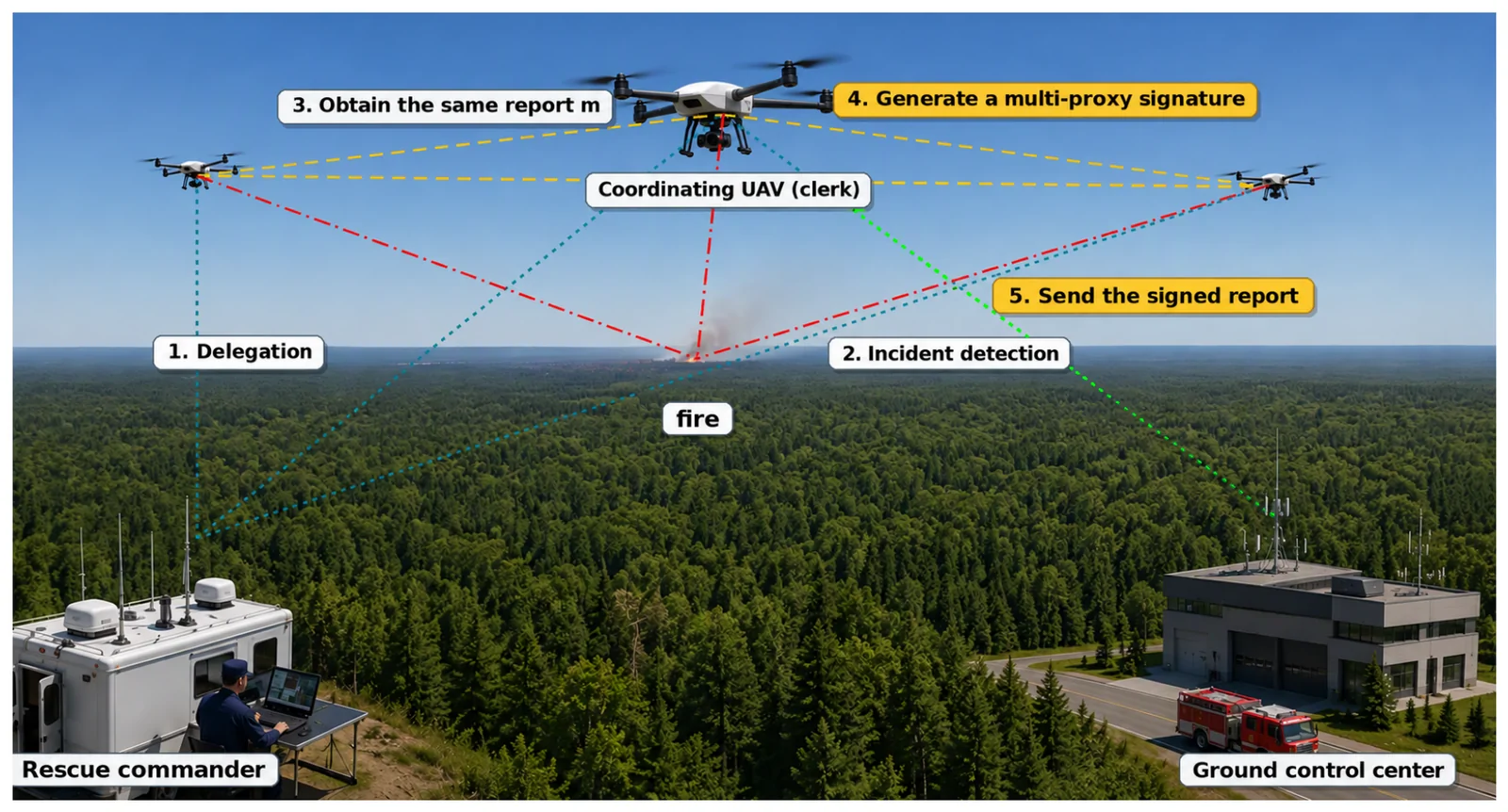
Application of IBMPS to UAV-assisted forest fire response.

Figure 1 illustrates this workflow. The rescue commander first prepares a warrant *w* specifying the mission identifier, the identities of all authorized UAVs, the mission objectives, the geographical scope, the authorization validity period, and the reporting requirements. The commander then generates a delegation that binds *w* to the ordered public-key list *L* of the designated UAVs and distributes the delegation and *L* to the authorized group.

Each designated UAV collects observations within the scope specified by *w*. To jointly endorse an incident report, the UAVs sign a common message *m* containing the mission identifier, the incident identifier, the incident location, the observation period, and a shared statement describing the incident. The signing procedure assumes that all designated UAVs have obtained the same encoded report *m*; report formation and distribution are outside the scope of the signature construction. Before signing, each UAV independently determines whether its observations support the report’s statement and whether the report satisfies the mission, geographical, temporal, and reporting constraints specified in *w*. Only when both conditions are satisfied does the UAV participate in the prescribed signing exchanges and generate a partial proxy signature on *m*.

A coordinating UAV, referred to as the clerk, collects and verifies the partial proxy signatures. The clerk belongs to the authorized group and also contributes its own partial proxy signature. After obtaining valid partial proxy signatures from all designated UAVs, the clerk aggregates them into a final multi-proxy signature and forwards the signed report to the ground control center. The center checks the report’s compliance with *w*, confirms that the declared UAV identities match those specified in the warrant, and verifies the multi-proxy signature using the corresponding public information. Acceptance provides authenticated evidence that the authorized group has jointly endorsed the report, thereby supporting disaster assessment and rescue decisions.

This workflow requires authentication of both the UAVs’ joint endorsement and the commander’s delegation of signing authority for the specified mission. Conventional digital signatures authenticate reports signed by individual UAVs, whereas aggregate signatures combine multiple signatures into a compact representation for aggregate verification. The unforgeability guarantees of an aggregate signature scheme depend on its underlying assumptions and the conditions imposed by its security model, including any key-registration requirements. Signature aggregation alone, however, does not establish that the rescue commander has authorized the participating UAVs to endorse reports for a particular mission within a specified validity period. An aggregate-signature-based solution therefore requires an additional mechanism to establish and verify this authorization.

IBMPS incorporates explicit delegation into the joint signing process, allowing the designated UAVs to collectively sign a common rescue report under the commander’s warrant. By verifying the resulting signature and checking the report and designated signer group against the warrant, the ground control center can authenticate the group’s joint endorsement under the specified delegation. This integration of delegated signing authority and joint report authentication motivates the use of IBMPS in UAV-assisted rescue missions.

### 1.1. Related Work

Proxy signatures (PS), introduced by Mambo et al. [5], allow an original signer to delegate signing authority to a proxy signer, who subsequently signs on the original signer’s behalf. Boldyreva et al. [6] established formal security foundations for this primitive by defining security requirements and presenting provably secure constructions. Malkin et al. [7] explored the relationship between proxy signatures and key-evolving signatures and developed a framework for hierarchical delegation. Schuldt et al. [8] extended the security treatment to explicitly address proxy signing-key exposure, emphasizing the need to distinguish the compromise of delegated signing keys from that of long-term private keys.

Subsequent research adapted proxy signatures to various cryptographic settings and delegation requirements. Zhang and Kim [9] developed identity-based blind and proxy signature constructions using bilinear pairings, while Lin and Jan [10] combined delegated signing with the blindness property to construct proxy blind signatures. Other extensions addressed revocation in multi-proxy signatures [11], proxy multi-signatures [12], and threshold proxy signatures [13,14,15]. These studies broadened the functionality of delegated signing to accommodate different authorization structures and operational requirements.

Multi-proxy signatures (MPS) extend signing delegation from an individual proxy to a group of cooperating proxy signers. Hwang and Shi [16] introduced a construction in which an original signer authorizes a proxy group to jointly sign on the original signer’s behalf without disclosing the original signer’s private key. Lee et al. [17] analyzed attacks against existing proxy signature schemes, developed stronger security classifications and constructions, and discussed applications to multiparty delegation. In a separate line of research, Qin et al. [18] investigated quantum multi-proxy signatures, which rely on quantum resources and signing mechanisms distinct from those of classical constructions.

Within the identity-based setting, Li and Chen [19] proposed pairing-based multi-proxy signature constructions and related variants. Their work demonstrated the integration of identity-based cryptography with collaborative delegation, although the security analysis did not include a formal reduction proof. Xu et al. [20] subsequently proposed an identity-based multi-proxy sequential signature scheme in which proxy signers contribute in a prescribed order. This construction supported sequential collaboration but also lacked a formal security proof. Cao and Cao [21] advanced the formal treatment of identity-based multi-proxy signatures (IBMPS) by defining a security model and presenting a construction with a security proof. However, Xiong et al. [22] later identified a forgery vulnerability in that construction and proposed a modification. This finding underscores the importance of distinguishing ordinary signatures from delegated signatures and accounting for their interaction in both scheme design and security analysis.

In parallel, Jin and Wen [23] introduced a certificateless multi-proxy signature (CLMPS) scheme with a formal security model capturing different adversarial capabilities. They analyzed its security under the computational Diffie–Hellman (CDH) assumption in the random oracle model (ROM). Although certificateless cryptography addresses the key-escrow issue inherent in identity-based systems, it introduces different key-management requirements and security assumptions. In the identity-based setting, Gu et al. [24] proposed an IBMPS construction in the standard model (SM), with a security framework covering self-proxy signing and proxy signing-key exposure. Its signature size and verification cost, however, grow linearly with the number of proxy signers, increasing the overhead for larger signing groups.

More recent work has emphasized computational efficiency and applications involving resource-constrained devices. Chen et al. [25] proposed an efficient IBMPS scheme with a security proof in the ROM. As noted by Shin et al. [26], the scheme generates partial signatures directly from long-term private keys and delegation information, without a separate randomized proxy signing-key generation procedure. This design raises further questions about the role of dedicated proxy signing keys in balancing signing efficiency and protection of long-term private keys.

Shin et al. [26] developed an IBMPS scheme for the Internet of Drones that incorporates randomized partial proxy signing keys. Each such key combines the proxy signer’s private key, the original signer’s delegation, and fresh randomness. Their construction supports partial signature verification and compact aggregation, and its security analysis accommodates proxy signing-key queries under the CDH assumption in the ROM. They also proposed an online/offline variant and evaluated computation time and energy consumption on a Raspberry Pi drone platform. Their surveillance and rescue scenarios demonstrate the practical relevance of delegated collaborative signing in UAV networks. Accordingly, randomized partial proxy signing keys, partial signature verification, compact aggregation, and UAV rescue applications constitute established capabilities on which subsequent IBMPS constructions can build.

The existing literature motivates further investigation of the trade-offs among delegation binding, proxy signing-key protection, security proof models, and computational efficiency. ROM proofs idealize hash functions as random oracles, whereas SM proofs avoid this idealization; this distinction alone does not establish that a ROM-based construction is insecure in practice. Taking Shin et al.’s scheme as the primary reference, our construction retains randomized proxy signing-key generation, partial signature verification, and compact aggregation while modifying the long-term key representation, the group-valued hash construction, and the binding of authorization context. Specifically, it uses an identity-associated private scalar together with an auxiliary public value, adopts Waters-style structured hashing based on collision-resistant hash functions, and binds delegation and report signing to the relevant mission and signer information. We refine the security model to account for signer collusion, proxy signing-key exposure, and released partial signatures. We establish a separate CDH-based reduction for each of the three forgery scenarios in Game 1, with explicit success-probability bounds and no public-key replacement. Finally, we estimate computational costs using a historical operation benchmark and compare the representation sizes of private keys, delegations, and signatures. These evaluations do not establish equivalent contemporary security levels or complete end-to-end communication costs.

### 1.2. Our Contributions

UAV-assisted rescue requires a designated group of UAVs to jointly endorse a common report under a commander’s mission-specific authorization. Meeting this requirement entails authenticating the participating UAVs, binding the delegation to the mission and designated signer group, and verifying their joint endorsement of the report. Building on the IBMPS scheme of Shin et al. [26], we retain randomized proxy signing-key generation, partial signature verification, and compact aggregation, while modifying the long-term key representation, hash construction, and authorization-context binding. Our main contributions are summarized as follows:

- **Mission- and Group-Bound Delegation.** We define a warrant *w* that specifies the mission identifier, authorized UAV identities, mission objectives, geographical scope, validity period, and reporting requirements. Whereas Shin et al. [26] use the delegation hash $H_{2}\left(w,R_{0}\right)$, our construction computes
  $$K_{OS}=H_{2}\left(w,ID_{OS},R_{OS},L\right),$$
  incorporating the original signer’s identity, auxiliary public value, and the ordered public-key list *L* of the designated UAVs. This binds the delegation to both the mission warrant and the selected proxy group. Before signing, each UAV checks the report against the warrant; before accepting the signed report, the ground control center checks report compliance and signer-group consistency. Together, these cryptographic bindings and application-level checks support reporting within the specified authorization scope.
- **Scalar-Based Long-Term Keys and Conditional Key-Recovery Analysis.** In contrast to the group-element representation of long-term private keys in Shin et al. [26], our construction uses an identity-associated private scalar together with an auxiliary public value. The corresponding private-key contribution to delegation and proxy-key generation takes the form $d_{i}E$. After verifying the delegation, each UAV derives its partial proxy signing key by combining the delegation, its private-key contribution, and fresh randomness:
  $$PSK_{PS_{i}}=u_{PS_{i}}K_{OS}+d_{PS_{i}}E+V_{OS}.$$
  This formulation preserves randomized proxy-key generation while allowing subsequent partial signature generation to use the derived proxy signing key. We also analyze long-term private-key recovery under the specified proxy-key exposure conditions. Recovering the target user’s private scalar yields a solution to CDH, provided that the target public key is honestly generated and remains unchanged.
- **Verifiable Aggregation with Authorization-Context Binding.** We retain the partial signature verification and compact aggregation supported by Shin et al. [26], while extending the hash inputs used for report signing. Instead of $H_{3}\left(m,X\right)$, all designated UAVs use the common value
  $$K_{PS}=H_{3}\left(m,w,ID_{OS},R_{OS},U_{MPS}\right),$$
  which incorporates the report, warrant, original signer’s information, and aggregate proxy-key public value into the signing context. The coordinating UAV verifies each partial signature and rejects invalid contributions before aggregation. Once valid contributions from all designated UAVs have been obtained, they are combined into a final signature containing four group elements and the warrant, with the same representation size as the signature of Shin et al. Final verification requires a fixed number of pairings and scalar multiplications, although signer-list processing remains dependent on group size. The construction requires contributions from every designated UAV and does not provide threshold signing.
- Explicit Collusion Modeling and Standard-Model Reductions. Whereas Shin et al. [26] analyze security in the random oracle model, our analysis employs Waters-style group-valued hash functions defined by public parameters, together with collision-resistant hashing, in the standard model. We refine the security model to distinguish delegation forgery, forgery of an honest proxy’s participation by a malicious original signer colluding with the remaining proxies, and forgery of the original signer’s authorization by all colluding proxies. The model also tracks proxy signing-key exposure and releases partial signatures to define the freshness requirements for a valid forgery. We establish a separate CDH-based reduction for each forgery scenario in Game 1, which excludes public-key replacement. The reductions explicitly account for adaptive query simulation, target-identity guessing, coefficient events, and negligible collision-related loss.
- **Model-Based Evaluation of Computational Costs and Representation Sizes.** We evaluate the computational costs of delegation, proxy-key generation, signing, and final verification, as well as the representation sizes of private keys, delegations, and final signatures. Under the adopted operation counts and historical benchmark timings, the estimated total computational cost with ten proxy signers is 19.34–44.00% lower than that of five of the six comparison schemes; the estimated final verification cost is 50.26–74.06% lower than that of the six schemes. Relative to Shin et al. [26], the corresponding estimated differences are 26.16% and 68.80%. Under the adopted encoding assumptions, the long-term private-key representation is 20 bytes, compared with 128 bytes in Shin et al.’s scheme [26]; the delegation and final signature representations are 276 bytes and 532 bytes, respectively. These are model-based comparisons, not implementation measurements or comparisons at verified equivalent contemporary security levels.

### 1.3. Organization

The remainder of this paper is organized as follows. Section 2 introduces the preliminary concepts required for the subsequent discussion. Section 3 presents the system model of the proposed scheme. Section 4 formalizes the corresponding security model. Section 5 describes the detailed construction of the proposed scheme. Section 6 provides the security proofs. Section 7 compares the proposed scheme with representative multi-proxy signature schemes in terms of security-related features, estimated computational costs, and key, delegation, and signature sizes. Finally, Section 8 summarizes the paper and draws the main conclusions.

## 2. Preliminaries

Let $G_{1}$ be an additive group of prime order *q*, and let $G_{2}$ be a multiplicative cyclic group of the same order. Let $0_{G_{1}}$ and $1_{G_{2}}$ denote the identity elements of $G_{1}$ and $G_{2}$, respectively. Consider a map $e:G_{1}\times G_{1}\to G_{2}$ satisfying the following properties:

Non-degeneracy: There exist $P,Q\in G_{1}$ such that $e\left(P,Q\right)\neq 1_{G_{2}}$.

Efficient computability: The value e(P,Q) can be computed efficiently for all $P,Q\in G_{1}$.

Bilinearity: For all $P,Q\in G_{1}$ and $\alpha ,\beta \in Z_{q}$, $e\left(\alpha P,\beta Q\right)=e{\left(P,Q\right)}^{\alpha \beta }$.

**Computational Diffie–Hellman (CDH) problem:** Given a generator *P* of $G_{1}$ and the elements αP and βP, where $\alpha ,\beta \in Z_{q}^{*}$ are unknown, compute αβP.
**CDH assumption:** No probabilistic polynomial-time algorithm solves the CDH problem with non-negligible probability.

A hash function family H=(HGen,HEval) consists of a probabilistic polynomial-time index generation algorithm HGen and a deterministic polynomial-time evaluation algorithm HEval. Given a security parameter μ, $HGen(1^{\mu })$ outputs a public index *s* that defines a function

$$H_{s}:{\left\{0,1\right\}}^{*}\to Y_{s},\;H_{s}\left(z\right)=HEval\left(s,z\right),$$

where $Y_{s}$ is the finite output space associated with *s*. The evaluation time is polynomial in μ and the input length.

The family *H* is collision resistant if, for every probabilistic polynomial-time adversary given *s*, the probability of finding distinct inputs $z,z^{\prime }$ such that $H_{s}\left(z\right)=H_{s}\left(z^{\prime }\right)$ is negligible in μ. This probability is taken over the randomness of both the index generation algorithm and the adversary.

The index *s* is public and is not a secret key. Once *s* is fixed, the hash evaluation is deterministic. In the standard model, hash functions are evaluated according to their public descriptions rather than modeled as random oracles.

## 3. The System Model of IBMPS

To facilitate understanding of the proposed scheme, Table 1 summarizes the principal notation used throughout this paper.

**Figure 2:**
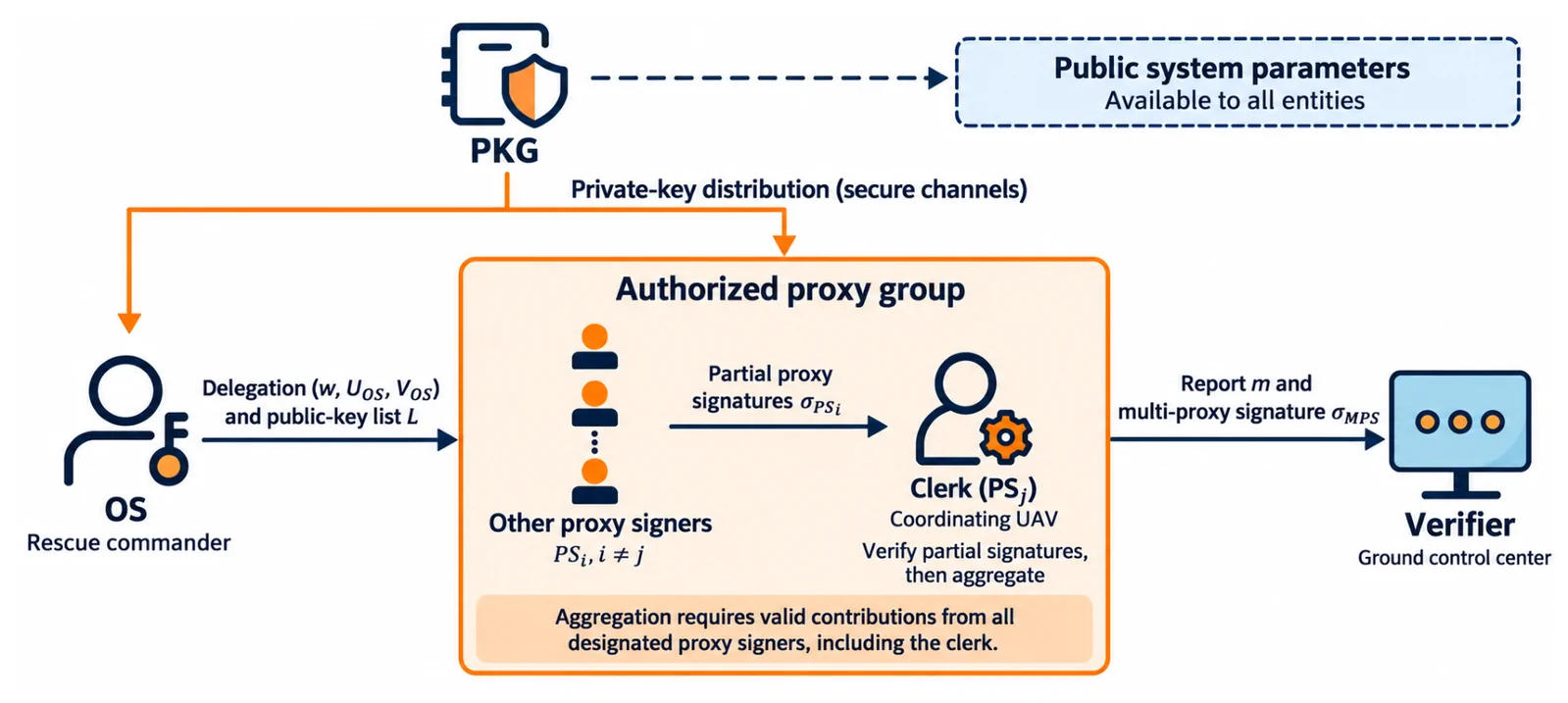
System architecture of the proposed IBMPS scheme.

The proposed IBMPS system comprises five functional roles: a private key generator (PKG), an original signer (OS), a group of proxy signers ${\left\{PS_{i}\right\}}_{i=1}^{n}$, a clerk, and a verifier. In the UAV-assisted rescue scenario considered in this paper, the PKG serves as a trusted key-management authority, the OS represents the rescue commander, and the proxy signers correspond to the UAVs assigned to the mission. The clerk is a coordinating UAV $PS_{j}$, where j∈{1,…,n}, selected from the proxy signer group and also acting as a proxy signer. The verifier represents the ground control center. Figure 2 illustrates the system architecture, and the responsibilities of each role are described below.

PKG: The PKG generates the system parameters and issues identity-associated private keys, together with the corresponding auxiliary public values, to the original signer and the proxy signers.

OS:The rescue commander OS specifies the mission identifier, the identities of all authorized UAVs, the mission objectives, the geographical scope, the authorization validity period, and the reporting requirements in a warrant *w*. Using its private key, the OS generates a delegation $\left(w,U_{OS},V_{OS}\right)$ bound to the designated proxy public-key list *L*. It then distributes the delegation and *L* to the authorized UAV group.

${\left\{PS_{i}\right\}}_{i=1}^{n}$: Each authorized UAV $PS_{i}$ verifies the received delegation and derives its partial proxy signing key from the delegation and its own private key. The UAV conducts observations within the authorized scope and assesses a common rescue report *m*, which specifies the mission identifier, the incident identifier, the incident location, the observation period, and a common incident statement. These five fields constitute the report *m* and are encoded in a fixed order using a canonical, unambiguous encoding. The mission identifier in *m* must match that specified in *w*. Each designated UAV participates in signing only if its observations support the statement and the report satisfies the constraints specified in *w*. It then completes the required signing exchanges, generates a partial proxy signature on *m*, and submits the resulting contribution to the clerk.

Clerk: The clerk is a coordinating UAV $PS_{j}$ selected from the proxy signer group. It participates in observation, report assessment, and signature generation. It additionally collects and verifies the partial proxy signatures on *m*. Once valid contributions have been obtained from all designated UAVs, including itself, the clerk constructs the final multi-proxy signature and forwards it, together with the report, to the ground control center.

Verifier: The ground control center checks that *m* satisfies the constraints specified in *w* and that the declared proxy identities and their corresponding public values match the authorized group. It then verifies the multi-proxy signature using the required public information. If all checks succeed, the report is accepted as jointly endorsed by the authorized UAV group and used to support rescue assessment and response decisions.

The proposed IBMPS scheme is defined by the following seven algorithms.

- **Setup (**μ**)**: Executed by the PKG, this algorithm takes a security parameter μ as input and generates the public system parameters params and the master secret key mk. The PKG publishes the system parameters params and keeps mk secret.
- **PriK-Extract (*****params*****,*****mk*****,** ${\mathrm{ID}}_{i}$**)**: Executed by the PKG, this algorithm takes the system parameters params, the master secret key mk, and a user identity $ID_{i}$ as input. It generates the corresponding private key $d_{i}$ and the corresponding auxiliary public value $R_{i}$. The PKG delivers $d_{i}$ through a secure channel and provides $R_{i}$ to the user.
- **PubK-Generate (**${\mathrm{ID}}_{i}$, $R_{i}$**)**: This algorithm takes a user identity $ID_{i}$ and the corresponding auxiliary public value $R_{i}$ as input. The user sets its public key to $PK_{i}=R_{i}$.
- **Dele-Generate (*****params*****,*****w*****,** ${\mathrm{ID}}_{\mathrm{OS}},{\mathrm{PK}}_{\mathrm{OS}},d_{\mathrm{OS}},L$**)**: Executed by the OS, this algorithm generates a delegation for a designated group of proxy signers. Before executing the algorithm, the OS constructs a warrant *w* specifying the mission identifier, the identities of all authorized proxy signers, the mission objectives, the geographical scope, the authorization validity period, and the reporting requirements. It also determines the ordered public-key list $L=(PK_{PS_{1}},PK_{PS_{2}},\cdots ,PK_{PS_{n}})$, where $PK_{PS_{i}}=R_{PS_{i}}$. The algorithm takes params, *w*, the original signer’s identity $ID_{OS}$, public key $PK_{OS}$, and private key $d_{OS}$, together with *L*, as input. It outputs the delegation $\left(w,U_{OS},V_{OS}\right)$, which the OS distributes alongside *L* to the designated proxy signers. Whenever *L* is included in a hash input, its entries follow the order of the corresponding proxy identities listed in *w* and are represented using a fixed, unambiguous encoding.
- **ProK-Generate (**params, $\left(w,U_{\mathrm{OS}},V_{\mathrm{OS}}\right)$, ${\mathrm{ID}}_{\mathrm{OS}}$, ${\mathrm{PK}}_{\mathrm{OS}}$, ${\mathrm{ID}}_{{\mathrm{PS}}_{i}}$, ${\mathrm{PK}}_{{\mathrm{PS}}_{i}}$, $d_{{\mathrm{PS}}_{i}}$, L**)**: This algorithm is executed by each proxy signer $PS_{i}$. It takes as input params, the delegation $\left(w,U_{OS},V_{OS}\right)$, the original signer’s identity $ID_{OS}$ and public key $PK_{OS}$, the proxy public-key list *L*, and the proxy signer’s own identity $ID_{PS_{i}}$, public key $PK_{PS_{i}}$, and private key $d_{PS_{i}}$. Before verifying the delegation, $PS_{i}$ first checks whether its own identity $ID_{PS_{i}}$ belongs to the proxy group specified by *w*, and confirms that its own public key $PK_{PS_{i}}$ matches the corresponding entry in *L*. If either condition is not satisfied, it outputs ⊥ and terminates. Then, $PS_{i}$ verifies the delegation $\left(w,U_{OS},V_{OS}\right)$. After successful verification, $PS_{i}$ derives its partial proxy signing key $PSK_{PS_{i}}$, and simultaneously generates and broadcasts the public value $U_{PS_{i}}$. The partial proxy signing key $PSK_{PS_{i}}$ is kept secret by $PS_{i}$ and is used to participate in the joint generation of multi-proxy signatures under the authorization specified in *w*; $U_{PS_{i}}$ is a public value used in partial signature verification and in computing $U_{MPS}$. The algorithm outputs $\left(PSK_{PS_{i}},U_{PS_{i}}\right)$, or ⊥ on failure.
- **MP-Sign** $\left(\mathrm{params},m,\left(w,U_{\mathrm{OS}},V_{\mathrm{OS}}\right),{\mathrm{ID}}_{\mathrm{OS}},{\mathrm{PK}}_{\mathrm{OS}},{\left\{{\mathrm{ID}}_{{\mathrm{PS}}_{i}}\right\}}_{i=1}^{n},{\left\{{\mathrm{PK}}_{{\mathrm{PS}}_{i}}\right\}}_{i=1}^{n},{\left\{{\mathrm{PSK}}_{{\mathrm{PS}}_{i}}\right\}}_{i=1}^{n},{\left\{U_{{\mathrm{PS}}_{i}}\right\}}_{i=1}^{n}\right)$: This distributed signing algorithm is jointly executed by the designated proxy signers, with $PS_{j}$ acting as the clerk. The collection ${\left\{PSK_{PS_{i}}\right\}}_{i=1}^{n}$ denotes the signers’ respective local secret inputs. The algorithm consists of two stages: (1) Partial signature generation. Each proxy signer $PS_{i}$ takes as input the system parameters params, the report *m*, the delegation $\left(w,U_{OS},V_{OS}\right)$, the original signer’s identity $ID_{OS}$ and public key $PK_{OS}$, the designated proxy group’s public information, and its own partial proxy signing key $PSK_{PS_{i}}$ and the public values ${\left\{U_{PS_{i}}\right\}}_{i=1}^{n}$ generated during ProK-Generate. For 1≤i≤n, $PS_{i}$ evaluates the common rescue report *m*, which contains the mission identifier, the incident identifier, the incident location, the observation period, and a common incident statement. Only when $PS_{i}$ verifies that *m* satisfies the constraints specified in *w* does $PS_{i}$ participate in the signing. It then outputs a partial proxy signature $\sigma_{PS_{i}}$ and transmits it to a designated proxy signer $PS_{j}$(j∈{1,2,⋯,n}), who serves as the clerk to aggregate the signatures. (2) Signature aggregation. The clerk collects and verifies the *n* partial proxy signatures $\left\{\sigma_{PS_{1}},\sigma_{PS_{2}},\cdots ,\sigma_{PS_{n}}\right\}$ from all proxy signers, and outputs the multi-proxy signature $\sigma_{MPS}$ on *m*.
- **MP-Verify** $\left(\mathrm{params},m,\sigma_{\mathrm{MPS}},{\mathrm{ID}}_{\mathrm{OS}},{\mathrm{PK}}_{\mathrm{OS}},{\left\{{\mathrm{ID}}_{{\mathrm{PS}}_{i}}\right\}}_{i=1}^{n},{\left\{{\mathrm{PK}}_{{\mathrm{PS}}_{i}}\right\}}_{i=1}^{n}\right)$: Executed by the verifier, this algorithm takes the system parameters params, the report *m*, the multi-proxy signature $\sigma_{MPS}$, the original signer’s identity $ID_{OS}$ and the public key $PK_{OS}$, the identities ${\left\{ID_{PS_{i}}\right\}}_{i=1}^{n}$ of the proxy signers ${\left\{PS_{i}\right\}}_{i=1}^{n}$, and their public keys ${\left\{PK_{PS_{i}}\right\}}_{i=1}^{n}$ as input. The verifier first extracts the warrant *w* from $\sigma_{MPS}$ and checks whether *m* satisfies its constraints. It checks that the supplied proxy identities correspond one-to-one to those specified in *w*, constructs the ordered public-key list *L* from the corresponding supplied public keys, and uses this list to compute $K_{OS}$ for aggregate signature verification. Finally, it verifies whether $\sigma_{MPS}$ is a valid multi-proxy signature on *m*, generated under *w* by the designated proxy signer group on behalf of the OS. The algorithm accepts if all checks succeed and rejects otherwise.

## 4. Security Model

The IBMPS system comprises one original signer and *n* proxy signers. We consider both collusion among proxy signers and collusion between the original signer and a subset of the proxy signers. The adversary A is allowed to adaptively corrupt participants and obtain their long-term private keys. For a target forgery, A may corrupt at most *n* of the n+1 participants, subject to the requirement that at least one participant whose authorization or signature is being forged remains uncorrupted.

We denote the identity of such a participant by $ID^{⋄}$. This requirement rules out the trivial case in which A obtains all long-term private keys needed to generate the target delegation or signature directly. Game 1 imposes additional restrictions on proxy signing-key queries and signing queries to exclude trivial forgeries derived from keys or signatures obtained through these queries.

The identity $ID^{⋄}$ is determined by the adversary’s final forgery and need not be fixed before the interaction. Depending on the forgery scenario, $ID^{⋄}$ corresponds to either the original signer or a proxy signer. Under this adversarial model, we distinguish the following three forgery scenarios.

- **Delegation forgery.** The adversary A impersonates an honest original signer with identity $ID^{⋄}$ and produces a valid delegation $\left(w^{*},U_{OS}^{*},V_{OS}^{*}\right)$, although the original signer has not authorized the designated proxy group under $w^{*}$. The adversary may have obtained delegations from the same original signer for other warrants or proxy groups.
- **Signature forgery without an honest proxy signer’s participation.** A malicious original signer colludes with n−1 corrupted proxy signers to produce a valid multi-proxy signature
  $$\sigma_{MPS}^{*}=\left(w^{*},U_{OS}^{*},U_{MPS}^{*},X_{MPS}^{*},V_{MPS}^{*}\right)$$
  on a report $m^{*}$. The signature purports to represent the participation of all *n* designated proxy signers, although the remaining honest proxy signer, with identity $ID^{⋄}$, has not signed $m^{*}$ under the target authorization. The adversary has not obtained this signer’s long-term private key or its partial proxy signing key for the target authorization. It may, however, have requested signatures from the honest signer on other reports.
- **Signature forgery without the original signer’s authorization.** The adversary A corrupts all *n* proxy signers and produces a valid multi-proxy signature on a report $m^{*}$ under a warrant $w^{*}$, although the honest original signer, with identity $ID^{⋄}$, has not authorized the designated proxy group under $w^{*}$. Delegations obtained from this original signer for other warrants or proxy groups do not constitute authorization for the target signature.

**Figure 3:**
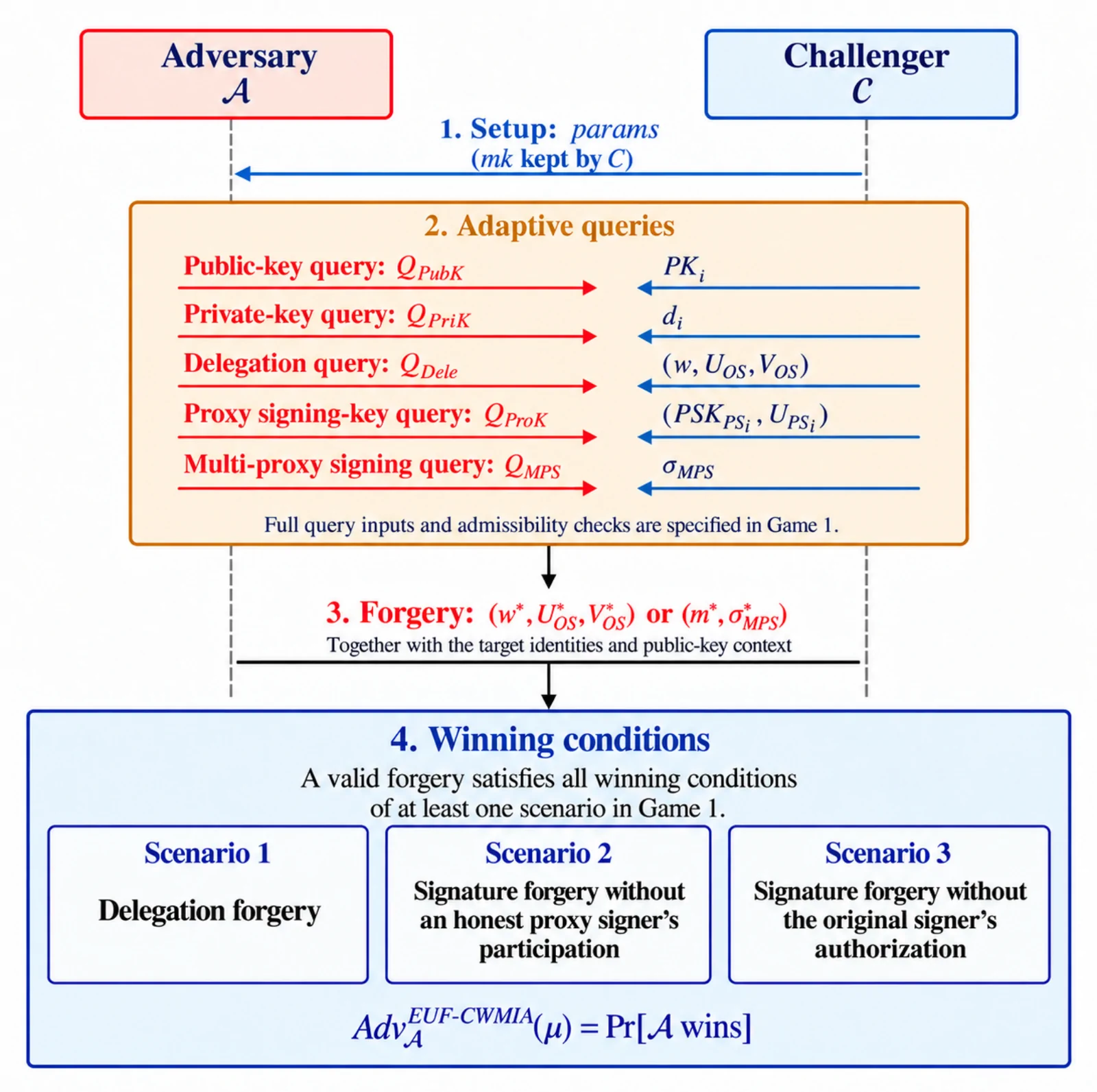
Overview of Game 1.

**Game 1.** We formalize the above forgery scenarios through the following game between a challenger C and a probabilistic polynomial-time adversary A. An overview of the game is provided in Figure 3.

**Setup phase.** The challenger C runs the Setup algorithm to generate the public system parameters params and the master secret key mk. It gives params to A and keeps mk secret.

**Query phase.** The adversary A may adaptively issue a polynomial number of the queries defined below. When a query involves an identity that has not yet been registered, the challenger C generates the corresponding key pair once, using PriK-Extract and PubK-Generate, and stores it for subsequent queries. The challenger’s internal use of a private key does not constitute disclosure of that key to A. Each identity is associated with a single public key generated by the challenger. Public-key replacement is not allowed in this game. The challenger maintains the following lists, all initially empty:

- $L_{\mathrm{Key}}$ stores tuples $\left(ID_{i},R_{i},d_{i}\right)$, where $\left(R_{i},d_{i}\right)$ is the key pair originally issued to $ID_{i}$.
- $L_{\mathrm{Pub}}$ stores tuples $\left(ID_{i},PK_{i}\right)$, where $PK_{i}$ is the registered public key associated with $ID_{i}$ and is initially set to $R_{i}$.
- $L_{\mathrm{Pri}}$ records each identity $ID_{i}$ for which $d_{i}$ has been returned to A.
- $L_{\mathrm{Dele}}$ stores tuples $\left(w,ID_{OS},PK_{OS},L,\left(w,U_{OS},V_{OS}\right)\right)$ for delegations returned to A, where $\left(w,U_{OS},V_{OS}\right)$ is the delegation on *w*.
- $L_{\mathrm{Pro}}$ stores tuples $\left(\left(w,U_{OS},V_{OS}\right),ID_{OS},PK_{OS},ID_{PS_{i}},PK_{PS_{i}},L,PSK_{PS_{i}},U_{PS_{i}}\right)$, where $PSK_{PS_{i}}$ is the generated partial proxy signing key of $PS_{i}$, and $U_{PS_{i}}$ is its corresponding public value.
- $L_{\mathrm{Exp}}$ stores tuples $\left(\left(w,U_{OS},V_{OS}\right),ID_{OS},PK_{OS},ID_{PS_{i}},PK_{PS_{i}},L,PSK_{PS_{i}},U_{PS_{i}}\right)$ for partial proxy signing keys returned to A through $Q_{\mathrm{ProK}}$.
- $L_{\mathrm{Sign}}$ stores a tuple $\left(m,\left(w,U_{OS},V_{OS}\right),ID_{OS},PK_{OS},{\left\{ID_{PS_{i}}\right\}}_{i=1}^{n},{\left\{PK_{PS_{i}}\right\}}_{i=1}^{n},L,\sigma_{MPS}\right)$ for each multi-proxy signature returned to A. It also stores a tuple $\left(m,\left(w,U_{OS},V_{OS}\right),ID_{OS},PK_{OS},ID_{PS_{i}},PK_{PS_{i}},L,\sigma_{PS_{i}}\right)$ for each partial signature released by an honest proxy signer. Both signature forms include the warrant *w*, which identifies the designated proxy signers.

In the reduction, C additionally maintains a reduction-only list $L_{\mathrm{Sim}}$ for proxy signing keys represented implicitly during signing-query simulations. Each entry records

$$(\left(w,U_{OS},V_{OS}\right),ID_{OS},PK_{OS},ID_{PS_{i}},PK_{PS_{i}},L,u_{PS_{i}},U_{PS_{i}}),$$

where $u_{PS_{i}}$ and $U_{PS_{i}}$ are the proxy signer’s random scalar and corresponding public value. The corresponding $PSK_{PS_{i}}$ is not stored because C cannot compute it explicitly. Entries in $L_{\mathrm{Sim}}$ are internal simulation state: they are not returned to A and are not recorded in $L_{\mathrm{Exp}}$.

**Public-key query** $Q_{\mathrm{PubK}}\left({\mathrm{ID}}_{i}\right)$**.** On receiving an identity $ID_{i}$, the challenger C returns its current public key $PK_{i}$ from $L_{\mathrm{Pub}}$. Initially, $PK_{i}=R_{i}$, where $R_{i}$ is generated during private key extraction.

**Private-key query** $Q_{\mathrm{PriK}}\left({\mathrm{ID}}_{i}\right)$**.** On receiving an identity $ID_{i}$, the challenger C returns the registered private key $d_{i}$ from $L_{\mathrm{Key}}$ and records $ID_{i}$ in $L_{\mathrm{Pri}}$ if it is not yet recorded.

**Delegation query** $Q_{\mathrm{Dele}}\left(w,{\mathrm{ID}}_{\mathrm{OS}},{\mathrm{PK}}_{\mathrm{OS}},L\right)$**.** The adversary A submits the warrant *w*, the original signer’s identity $ID_{OS}$ and public key $PK_{OS}$, and the public-key list *L* of all designated proxy signers. The challenger C checks that the supplied public keys match the current public keys of their corresponding identities and that *L* is consistent with the proxy group specified in *w*. If either check fails, it returns ⊥. Otherwise, it uses the corresponding original signer’s private key internally to run Dele-Generate. The challenger C returns the delegation $\left(w,U_{OS},V_{OS}\right)$ together with *L* and records $\left(w,ID_{OS},PK_{OS},L,\left(w,U_{OS},V_{OS}\right)\right)$ in $L_{\mathrm{Dele}}$.

**Proxy signing-key query** $Q_{\mathrm{ProK}}\left(\left(w,U_{\mathrm{OS}},V_{\mathrm{OS}}\right),{\mathrm{ID}}_{\mathrm{OS}},{\mathrm{PK}}_{\mathrm{OS}},{\mathrm{ID}}_{{\mathrm{PS}}_{i}},{\mathrm{PK}}_{{\mathrm{PS}}_{i}},L\right)$**.** The adversary A submits a delegation $\left(w,U_{OS},V_{OS}\right)$, the original signer’s identity $ID_{OS}$ and public key $PK_{OS}$, a proxy signer’s identity $ID_{PS_{i}}$ and public key $PK_{PS_{i}}$, and the designated proxy public-key list *L*. The challenger C verifies the delegation, checks the supplied public keys, and confirms that $ID_{PS_{i}}$ belongs to the proxy group specified in *w*. If any check fails, it returns ⊥. Otherwise, the challenger proceeds as follows:

(1) If $L_{\mathrm{Pro}}$ contains an entry matching the delegation, the original signer, the proxy signer, and the designated proxy group, including their public keys, the challenger retrieves the stored $PSK_{PS_{i}}$ and $U_{PS_{i}}$.
(2) Otherwise, the challenger uses the corresponding private key $d_{PS_{i}}$ internally to run ProK-Generate and obtains $PSK_{PS_{i}}=u_{PS_{i}}K_{OS}+d_{PS_{i}}E+V_{OS},\;U_{PS_{i}}=u_{PS_{i}}P$. It stores these values, together with the associated delegation, identities, and public keys, in $L_{\mathrm{Pro}}$.
(3) The challenger returns $\left(PSK_{PS_{i}},U_{PS_{i}}\right)$ to the adversary A and records the corresponding key exposure in $L_{\mathrm{Exp}}$. Its internal use of $d_{PS_{i}}$ does not constitute disclosure of that private key.

**Multi-proxy signing query** $Q_{\mathrm{MPS}}\left(m,\left(w,U_{\mathrm{OS}},V_{\mathrm{OS}}\right),{\mathrm{ID}}_{\mathrm{OS}},{\mathrm{PK}}_{\mathrm{OS}},{\left\{{\mathrm{ID}}_{{\mathrm{PS}}_{i}}\right\}}_{i=1}^{n},{\left\{{\mathrm{PK}}_{{\mathrm{PS}}_{i}}\right\}}_{i=1}^{n}\right)$**.** The challenger C verifies the delegation, checks that *m* satisfies the conditions in *w*, and confirms that the supplied public keys match $L_{\mathrm{Pub}}$ and the designated proxy group. If any check fails, it returns ⊥. Otherwise, it carries out the following steps:

(1) *Partial signature generation.* For each proxy signer $PS_{i}$, the challenger C retrieves a matching partial proxy signing key from $L_{\mathrm{Pro}}$. If no matching entry exists, it obtains $d_{PS_{i}}$ from $L_{\mathrm{Key}}$, runs ProK-Generate internally, and stores the resulting key and public value in $L_{\mathrm{Pro}}$.
  The challenger C then performs the partial signing steps of MP-Sign, ensuring that all participating signers use the same $U_{MPS}$ and $K_{PS}$. For each honest signer whose partial signature is released, it records $\left(m,\left(w,U_{OS},V_{OS}\right),ID_{OS},PK_{OS},ID_{PS_{i}},PK_{PS_{i}},L,\sigma_{PS_{i}}\right)$ in $L_{\mathrm{Sign}}$. If the adversary A controls some participants, the challenger C performs only the honest signers’ steps and exchanges the prescribed public messages and partial signatures with the adversary A. Records of released partial signatures are retained even if aggregation is not completed.
(2) *Verification and aggregation.* Once all partial signatures are available, the challenger C verifies them according to MP-Sign. If any partial signature is invalid, it returns ⊥. Otherwise, it aggregates them into $\sigma_{MPS}=\left(w,U_{OS},U_{MPS},X_{MPS},V_{MPS}\right)$, returns $\sigma_{MPS}$ to the adversary A, and records $\left(m,\left(w,U_{OS},V_{OS}\right),ID_{OS},PK_{OS},{\left\{ID_{PS_{i}}\right\}}_{i=1}^{n},{\left\{PK_{PS_{i}}\right\}}_{i=1}^{n},L,\sigma_{MPS}\right)$ in $L_{\mathrm{Sign}}$.

The challenger’s internal generation or use of secret keys does not constitute key disclosure. Queries involving a participant who remains honest in the final forgery are permitted, subject to the winning conditions below.

**Forgery phase.** Before evaluating the winning conditions, the challenger checks that all identities in the final output are registered and that their supplied public keys match the corresponding entries in $L_{\mathrm{Pub}}$. It also checks that the proxy identities are distinct and correspond exactly, in the prescribed order, to those specified in $w^{*}$. If any check fails, the adversary loses. The adversary A outputs the target original signer’s identity and public key, the identities and public keys of the designated proxy signers, and either a delegation $\left(w^{*},U_{OS}^{*},V_{OS}^{*}\right)$ or a report $m^{*}$ accompanied by a multi-proxy signature $\sigma_{MPS}^{*}=\left(w^{*},U_{OS}^{*},U_{MPS}^{*},X_{MPS}^{*},V_{MPS}^{*}\right)$. The adversary A wins if all conditions of at least one of the following scenarios are satisfied.

**Scenario 1: Delegation forgery.** The adversary A impersonates an original signer with identity $ID_{OS}^{*}$ and outputs $\left(w^{*},U_{OS}^{*},V_{OS}^{*}\right)$ such that:

(1) The delegation passes verification for the specified original signer and designated proxy group.
(2) $ID_{OS}^{*}\notin L_{\mathrm{Pri}}$; hence, the adversary A has not obtained the original signer’s long-term private key through $Q_{\mathrm{PriK}}\left(ID_{OS}^{*}\right)$.
(3) $L_{\mathrm{Dele}}$ contains no delegation issued for the same original signer identity and public key, warrant $w^{*}$, and designated proxy identities and public keys.

**Scenario 2: Signature forgery without an honest proxy signer’s participation.** The adversary A corrupts the original signer and n−1 proxy signers and outputs $\sigma_{MPS}^{*}$ on $m^{*}$ under $w^{*}$. Without loss of generality, let $PS_{1}$ be the remaining honest proxy signer. The adversary A wins if:

(1) MP-Verify accepts $\sigma_{MPS}^{*}$ on $m^{*}$.
(2) $ID_{PS_{1}}^{*}\notin L_{\mathrm{Pri}}$; hence, the adversary has not obtained this signer’s long-term private key through a private-key query.
(3) $L_{\mathrm{Exp}}$ contains no record showing that the adversary A obtained $PS_{1}$’s partial proxy signing key for the target warrant, original signer identity and public key, and designated proxy identities and public keys.
(4) $L_{\mathrm{Sign}}$ contains no record of $PS_{1}$ signing $m^{*}$ under the same warrant, original signer identity and public key, and designated proxy identities and public keys. This condition applies to both released partial signatures and returned multi-proxy signatures.

**Scenario 3: Signature forgery without the original signer’s authorization.** The adversary A corrupts all *n* proxy signers and outputs $\sigma_{MPS}^{*}$ on $m^{*}$ under $w^{*}$, claiming authorization from an original signer with identity $ID_{OS}^{*}$. The adversary A wins if:

(1) MP-Verify accepts $\sigma_{MPS}^{*}$ on $m^{*}$.
(2) $ID_{OS}^{*}\notin L_{\mathrm{Pri}}$; hence, the adversary A has not obtained the original signer’s long-term private key through $Q_{\mathrm{PriK}}\left(ID_{OS}^{*}\right)$.
(3) $L_{\mathrm{Dele}}$ contains no delegation issued for the same original signer identity and public key, warrant $w^{*}$, and designated proxy identities and public keys.
(4) $L_{\mathrm{Sign}}$ contains no multi-proxy signature returned on $m^{*}$ under the same warrant, original signer identity and public key, and designated proxy identities and public keys.

These winning conditions permit queries concerning other reports and authorizations, subject to the stated private-key and proxy signing-key restrictions. We denote existential unforgeability against adaptive chosen-warrant, chosen-message, and chosen-identity attacks by EUF-CWMIA. The adversary’s advantage is its probability of winning Game 1:

$$Adv_{A}^{EUF-CWMIA}\left(\mu \right)=\mathrm{Pr}\left[A\mathrm{wins}\right].$$

The scheme is secure in this model if the advantage is negligible in μ for every probabilistic polynomial-time adversary A.

## 5. Scheme Construction

The proposed IBMPS scheme comprises seven algorithms: Setup, PriK-Extract, PubK-Generate, Dele-Generate, ProK-Generate, MP-Sign, and MP-Verify. The original signer first issues a delegation that binds the authorization warrant *w* to the designated proxy signers. Upon validating the delegation, each proxy signer derives a partial proxy signing key using its private key and fresh randomness. To sign a common report *m*, the proxy signers generate partial signatures using a common hash value $K_{PS}$. A designated clerk verifies these contributions and aggregates them into a multi-proxy signature. The correctness of the aggregate verification equation follows from the signature construction and the bilinearity of the pairing.

- **Setup:** Given a security parameter μ, the PKG selects an additive cyclic group $G_{1}$ and a multiplicative cyclic group $G_{2}$ of the same prime order $q>2^{\mu }$, together with an efficiently computable, non-degenerate bilinear pairing
  $$e:G_{1}\times G_{1}\to G_{2}.$$

Let *P* be a generator of $G_{1}$. The PKG samples *E* uniformly from $G_{1}\setminus \left\{0_{G_{1}}\right\}$ and independently samples $T_{0},\ldots ,T_{\ell },S_{0},\ldots ,S_{\ell }$ uniformly from $G_{1}$, where ℓ=ℓ(μ) is a polynomially bounded positive integer.

It also samples $x\in Z_{q}^{*}$, sets the master secret key to mk=x, and computes $P_{pub}=xP$.

The PKG specifies the public indices of

$$H_{1}:{\left\{0,1\right\}}^{*}\to Z_{q}^{*},\;{\hat{H}}_{2},{\hat{H}}_{3}:{\left\{0,1\right\}}^{*}\to {\left\{0,1\right\}}^{\ell },$$

where ${\hat{H}}_{2}$ and ${\hat{H}}_{3}$ are independently selected from collision-resistant hash families. Their indices are omitted from the notation for brevity.

For an input *z*, let

$$\left(z_{1},\ldots ,z_{\ell }\right)={\hat{H}}_{2}\left(z\right),\;\left(z_{1}^{\prime },\ldots ,z_{\ell }^{\prime }\right)={\hat{H}}_{3}\left(z\right).$$

The group-valued functions $H_{2},H_{3}:{\left\{0,1\right\}}^{*}\to G_{1}$ are defined by

$$H_{2}\left(z\right)=T_{0}+\sum_{j=1}^{\ell }z_{j}T_{j},\;H_{3}\left(z\right)=S_{0}+\sum_{j=1}^{\ell }z_{j}^{\prime }S_{j}.$$

All multipart hash inputs are encoded canonically and unambiguously, with field boundaries and list ordering preserved. In particular, the proxy public-key list is represented as the ordered tuple

$$L=(PK_{PS_{1}},PK_{PS_{2}},\ldots ,PK_{PS_{n}}),$$

whose entries follow the order of the corresponding proxy identities specified in the warrant *w*.

The PKG publishes

$$params=(G_{1},G_{2},q,e,P,E,P_{pub},{\left\{T_{j}\right\}}_{j=0}^{\ell },{\left\{S_{j}\right\}}_{j=0}^{\ell },H_{1},{\hat{H}}_{2},{\hat{H}}_{3}),$$

including the public hash indices and function descriptions, and keeps the master secret key mk=x secret. The functions $H_{2}$ and $H_{3}$ are evaluated directly from these public parameters.

- **PriK-Extract:** For a user with identity $ID_{i}$, the PKG samples $r_{i}\in Z_{q}^{*}$ and computes
  $$R_{i}=r_{i}P,\;h_{i}=H_{1}\left(ID_{i},R_{i}\right),\;d_{i}=\left(r_{i}+h_{i}x\right)\;\mathrm{mod}\;q.$$

The PKG sends $R_{i}$ to the user and delivers the private key $d_{i}$ through a secure channel. The user recomputes $h_{i}=H_{1}\left(ID_{i},R_{i}\right)$ and accepts $d_{i}$ if and only if

$$d_{i}P=R_{i}+h_{i}P_{pub}.$$

Otherwise, the user rejects the key.

- **PubK-Generate:** The user with identity $ID_{i}$ sets $PK_{i}=R_{i}$, where $R_{i}$ is obtained from the PKG during PriK-Extract. After this step, the original signer OS holds the public-private key pair $\left(R_{OS},d_{OS}\right)$, and each proxy signer $PS_{i}$ holds the public-private key pair $\left(R_{PS_{i}},d_{PS_{i}}\right)$, for i=1,…,n.
- **Dele-Generate:** The original signer OS specifies an authorization warrant *w* and the ordered public-key list
  $$L=(PK_{PS_{1}},PK_{PS_{2}},\ldots ,PK_{PS_{n}})$$
  of the designated proxy signers. The warrant specifies the mission identifier, the identities of all authorized proxy signers, the mission objectives, the geographical scope, the authorization validity period, and the reporting requirements.

The original signer samples $u_{OS}\in Z_{q}^{*}$ and computes

$$\begin{array}{cc} U_{OS} & =u_{OS}P,\\ K_{OS} & =H_{2}\left(w,ID_{OS},R_{OS},L\right),\\ V_{OS} & =d_{OS}E+u_{OS}K_{OS}. \end{array}$$

It sends the delegation $\left(w,U_{OS},V_{OS}\right)$ and the ordered list *L* to each designated proxy signer $PS_{i}$. The input to $H_{2}$ binds the delegation to the warrant, the original signer’s identity and public value, and the ordered public-key list of the designated proxy group.

- **ProK-Generate:** Before verifying the delegation, $PS_{i}$ checks that $ID_{PS_{i}}$ belongs to the proxy group specified in *w* and that $PK_{PS_{i}}$ matches the corresponding entry in *L*. If either check fails, it returns ⊥.

The proxy signer computes

$$K_{OS}=H_{2}\left(w,ID_{OS},R_{OS},L\right),\;h_{OS}=H_{1}\left(ID_{OS},R_{OS}\right),$$

and verifies the delegation by checking

$$e\left(V_{OS},P\right)=e\left(E,R_{OS}+h_{OS}P_{pub}\right)e\left(K_{OS},U_{OS}\right).$$

If verification fails, $PS_{i}$ returns ⊥. Otherwise, it samples $u_{PS_{i}}\in Z_{q}^{*}$ and computes

$$U_{PS_{i}}=u_{PS_{i}}P,\;PSK_{PS_{i}}=u_{PS_{i}}K_{OS}+d_{PS_{i}}E+V_{OS}.$$

The partial proxy signing key $PSK_{PS_{i}}$ combines the verified delegation, the proxy signer’s private-key contribution, and fresh randomness. The signer broadcasts $U_{PS_{i}}$ to the proxy group and keeps $PSK_{PS_{i}}$ secret for subsequent partial signature generation. The public value $U_{PS_{i}}$ is used in partial signature verification and in computing

$$U_{MPS}=\sum_{i=1}^{n}U_{PS_{i}}.$$

The algorithm outputs $\left(PSK_{PS_{i}},U_{PS_{i}}\right)$, or ⊥ on failure.

- **MP-Sign**: To sign a common rescue report *m* on behalf of the original signer OS under the warrant *w*, all designated proxy signers generate partial proxy signatures. A clerk $PS_{j}$, where j∈{1,…,n}, verifies and aggregates their contributions. The clerk also contributes its own partial proxy signature.

*(1) Partial signature generation* Each proxy signer $PS_{i}$ first checks whether its local observation supports the common statement in the report *m* and whether *m* satisfies the authorization conditions in *w*. If either check fails, it returns ⊥.

Using the public values broadcast during ProK-Generate, each proxy signer computes

$$U_{MPS}=\sum_{i=1}^{n}U_{PS_{i}},\;K_{PS}=H_{3}\left(m,w,ID_{OS},R_{OS},U_{MPS}\right).$$

This step requires the public values of all designated proxy signers to be available. All participating signers use the same report *m*, warrant *w*, and aggregate value $U_{MPS}$.

The proxy signer samples $x_{PS_{i}}\in Z_{q}^{*}$ and computes

$$X_{PS_{i}}=x_{PS_{i}}P,\;V_{PS_{i}}=x_{PS_{i}}K_{PS}+PSK_{PS_{i}}.$$

It sends the partial proxy signature

$$\sigma_{PS_{i}}=\left(w,U_{OS},V_{OS},X_{PS_{i}},V_{PS_{i}}\right)$$

and its public key $R_{PS_{i}}$ to the clerk.

*(2) Partial signature verification and aggregation*

The clerk uses the ordered public-key list

$$L=(PK_{PS_{1}},PK_{PS_{2}},\ldots ,PK_{PS_{n}})$$

specified in the delegation. It checks that each received contribution corresponds to a designated proxy signer and that the supplied public key matches the corresponding entry in *L*. If either check fails, it returns ⊥.

Using the public values of all designated proxy signers, the clerk recomputes

$$\begin{array}{cc} U_{MPS} & =\sum_{i=1}^{n}U_{PS_{i}},\\ K_{OS} & =H_{2}\left(w,ID_{OS},R_{OS},L\right),\\ h_{OS} & =H_{1}\left(ID_{OS},R_{OS}\right),\\ K_{PS} & =H_{3}\left(m,w,ID_{OS},R_{OS},U_{MPS}\right),\\ h_{PS_{i}} & =H_{1}\left(ID_{PS_{i}},R_{PS_{i}}\right),\;i=1,\ldots ,n. \end{array}$$

For each i∈{1,…,n}, including its own contribution, the clerk checks

$$\begin{array}{cc} e(V_{PS_{i}},P)= & e\left(E,R_{OS}+R_{PS_{i}}+\left(h_{OS}+h_{PS_{i}}\right)P_{pub}\right)\\ & \cdot e\left(X_{PS_{i}},K_{PS}\right)\cdot e\left(U_{OS}+U_{PS_{i}},K_{OS}\right). \end{array}$$

This verification equation follows by expanding the partial signature as

$$V_{PS_{i}}=x_{PS_{i}}K_{PS}+\left(u_{PS_{i}}+u_{OS}\right)K_{OS}+\left(d_{PS_{i}}+d_{OS}\right)E$$

and applying the private-key relations and the bilinearity of the pairing.

If any verification fails, the clerk returns ⊥. Aggregation is performed only after one valid partial signature has been obtained from every designated proxy signer, including the clerk. If any required contribution is unavailable, the joint signature cannot be completed. The algorithm does not permit aggregation over a subset of the designated proxy group.

Once all required partial signatures have been verified, the clerk computes

$$V_{MPS}=\sum_{i=1}^{n}V_{PS_{i}},\;X_{MPS}=\sum_{i=1}^{n}X_{PS_{i}},$$

and outputs the multi-proxy signature

$$\sigma_{MPS}=\left(w,U_{OS},U_{MPS},X_{MPS},V_{MPS}\right)$$

for the report *m*.

Since all partial signatures use the same $K_{PS}$ and $K_{OS}$, their corresponding terms can be added in $G_{1}$. Each partial signature contains one occurrence of $V_{OS}$; therefore, $V_{MPS}$ contains $nV_{OS}$. This determines the coefficients of the original signer’s terms in the aggregate verification equation.

- **MP-Verify**: Given the report *m*, the original signer’s identity–public-key pair $\left(ID_{OS},R_{OS}\right)$, the proxy signers’ pairs ${\left\{\left(ID_{PS_{i}},R_{PS_{i}}\right)\right\}}_{i=1}^{n}$, and the multi-proxy signature
  $$\sigma_{MPS}=\left(w,U_{OS},U_{MPS},X_{MPS},V_{MPS}\right),$$
  the verifier checks whether *m* satisfies the conditions specified in *w*. It also checks that the supplied proxy identities are distinct and correspond exactly to those specified in *w*. It constructs the ordered public-key list
  $$L=(PK_{PS_{1}},PK_{PS_{2}},\ldots ,PK_{PS_{n}})$$
  according to the identity order in *w*, where $PK_{PS_{i}}=R_{PS_{i}}$. If any check fails, it rejects the signature.

Otherwise, the verifier computes

$$\begin{array}{cc} K_{OS} & =H_{2}\left(w,ID_{OS},R_{OS},L\right),\\ h_{OS} & =H_{1}\left(ID_{OS},R_{OS}\right),\\ K_{PS} & =H_{3}\left(m,w,ID_{OS},R_{OS},U_{MPS}\right),\\ h_{PS_{i}} & =H_{1}\left(ID_{PS_{i}},R_{PS_{i}}\right),\;i=1,\ldots ,n. \end{array}$$

The list *L* uses the same ordering and canonical encoding convention as in Dele-Generate. The verifier accepts $\sigma_{MPS}$ if and only if

$$\begin{array}{cc} e(V_{MPS},P) & =e(K_{PS},X_{MPS})\\ & \;\cdot e\left(E,\sum_{i=1}^{n}R_{PS_{i}}+nR_{OS}+\left(\sum_{i=1}^{n}h_{PS_{i}}+nh_{OS}\right)P_{pub}\right)\\ & \;\cdot e(K_{OS},nU_{OS}+U_{MPS}). \end{array}$$

Otherwise, it rejects the signature.

The correctness of the scheme follows from the definition of $V_{PS_{i}}$:

$$V_{PS_{i}}=x_{PS_{i}}K_{PS}+\left(u_{PS_{i}}+u_{OS}\right)K_{OS}+\left(d_{PS_{i}}+d_{OS}\right)E.$$

Using bilinearity and the private-key relations

$$d_{OS}P=R_{OS}+h_{OS}P_{pub},\;d_{PS_{i}}P=R_{PS_{i}}+h_{PS_{i}}P_{pub},$$

we obtain

$$\begin{array}{cc} e(V_{MPS},P)\; & =e\left(\sum_{i=1}^{n}V_{PS_{i}},P\right)\\ \; & =e\left(\sum_{i=1}^{n}\left(x_{PS_{i}}K_{PS}+PSK_{PS_{i}}\right),P\right)\\ \; & =e\left(\sum_{i=1}^{n}x_{PS_{i}}K_{PS},P\right)\\ \; & \;\cdot e\left(\sum_{i=1}^{n}\left(\left(u_{PS_{i}}+u_{OS}\right)K_{OS}+\left(d_{PS_{i}}+d_{OS}\right)E\right),P\right)\\ \; & =e\left(K_{PS},\sum_{i=1}^{n}X_{PS_{i}}\right)\cdot e\left(K_{OS},nU_{OS}+\sum_{i=1}^{n}U_{PS_{i}}\right)\\ \; & \;\cdot e\left(E,\sum_{i=1}^{n}R_{PS_{i}}+nR_{OS}+\left(\sum_{i=1}^{n}h_{PS_{i}}+nh_{OS}\right)P_{pub}\right)\\ \; & =e\left(K_{PS},X_{MPS}\right)\cdot e\left(K_{OS},nU_{OS}+U_{MPS}\right)\\ \; & \;\cdot e\left(E,\sum_{i=1}^{n}R_{PS_{i}}+nR_{OS}+\left(\sum_{i=1}^{n}h_{PS_{i}}+nh_{OS}\right)P_{pub}\right). \end{array}$$

Hence, every correctly generated multi-proxy signature satisfies the verification equation.

## 6. Security Proof

### 6.1. Security Proof of Unforgeability

**Theorem** **1.** *Under the CDH assumption in $G_{1}$ and the collision resistance of ${\hat{H}}_{2},{\hat{H}}_{3}$, the proposed IBMPS scheme is existentially unforgeable in Game 1, which permits adaptive queries and the specified collusion but excludes public-key replacement. For any PPT adversary A and each Scenario i, there exists a PPT CDH solver $C_{i}$ satisfying*

$$\varepsilon_{i}\leq L_{i}{\mathrm{Adv}}_{C_{i}}^{\mathrm{CDH}}+\mathrm{negl}\left(\mu \right),\;i\in \left\{1,2,3\right\},$$

*where $\varepsilon_{i}$ is the Scenario i winning probability, ${\mathrm{Adv}}_{C_{i}}^{\mathrm{CDH}}$ is the solver’s success probability, and*

$$\begin{array}{cc} L_{1} & =4q_{PU}Q_{1}\left(\ell +1\right),\\ L_{2} & =16q_{PU}Q_{1}Q_{2}{\left(\ell +1\right)}^{2},\\ L_{3} & =16q_{PU}Q_{1}{\left(\ell +1\right)}^{2}. \end{array}$$

*The query bounds are defined below, with $q>2\max\left\{Q_{1},Q_{2}\right\}\left(\ell +1\right)$, 1≤n<q, and $Q_{2}=1$ in Scenario 3. Simulation and extraction coefficient events are accounted for in $L_{i}$, not assumed separately.*

**Proof.**  Let A be a probabilistic polynomial-time adversary in Game 1. The reduction algorithm C receives a CDH instance (P,aP,bP) for the scheme’s group parameters, where a,b are independently and uniformly chosen from $Z_{q}^{*}$, and aims to compute abP.

**Setup Phase.** The reduction completes the setup phase in the following three steps.

*(1) Generates master key and public parameters.*

The reduction uniformly selects $x\in Z_{q}^{*}$ and sets

$$mk=x,\;P_{pub}=xP,\;E=bP.$$

It retains mk privately. Since *b* is uniform in $Z_{q}^{*}$, *E* is a uniformly random generator of $G_{1}$.

*(2) Generates Waters hash parameters.*

The reduction generates $H_{1}$, ${\hat{H}}_{2}$, and ${\hat{H}}_{3}$ as in the real Setup algorithm, with

$$H_{1}:{\left\{0,1\right\}}^{*}\to Z_{q}^{*},\;{\hat{H}}_{2},{\hat{H}}_{3}:{\left\{0,1\right\}}^{*}\to {\left\{0,1\right\}}^{\ell }.$$

Let $Q_{1},Q_{2}\geq 1$ be fixed polynomial upper bounds on the numbers of distinct inputs for which the simulation requires nonzero coefficients $J_{2}$ and $J_{3}$, respectively. In Scenario 2, $Q_{2}$ bounds all distinct $H_{3}$ inputs at which the target proxy releases a partial signature, including queries answered using explicit keys. This conservative set contains every input requiring nonzero $J_{3}$ in an implicit-key simulation. Repeated inputs are counted once. The bounds are fixed before the interaction. Set

$$M_{1}=2Q_{1},\;M_{2}=2Q_{2},$$

and assume

$$q>\max\left\{M_{1},M_{2}\right\}\left(\ell +1\right).$$

The reduction independently and uniformly samples

$$\begin{array}{cc} c_{0},\ldots ,c_{\ell } & \in \{0,\ldots ,M_{1}-1\},\\ c_{0}^{\prime },\ldots ,c_{\ell }^{\prime } & \in \{0,\ldots ,M_{2}-1\},\\ k_{1},k_{2} & \in \{0,\ldots ,\ell \}, \end{array}$$

and defines the integer coefficients

$$\begin{array}{cccc} \alpha_{0} & =c_{0}-M_{1}k_{1}, & \alpha_{j} & =c_{j},\;j=1,\ldots ,\ell ,\\ \gamma_{0} & =c_{0}^{\prime }-M_{2}k_{2}, & \gamma_{j} & =c_{j}^{\prime },\;j=1,\ldots ,\ell . \end{array}$$

It independently samples

$$\beta_{0},\ldots ,\beta_{\ell },\;\eta_{0},\ldots ,\eta_{\ell }\overset{\$}{\leftarrow }Z_{q}$$

and computes

$$T_{j}=\alpha_{j}E+\beta_{j}P,\;S_{j}=\gamma_{j}E+\eta_{j}P,\;j=0,\ldots ,\ell .$$

It does not resample if any $T_{j}$ or $S_{j}$ equals $0_{G_{1}}$. Since $\beta_{j}$ and $\eta_{j}$ are sampled independently and uniformly from $Z_{q}$, and *P* generates $G_{1}$, each $T_{j}$ and $S_{j}$ is uniformly distributed over $G_{1}$, including $0_{G_{1}}$, as in the Setup algorithm.

**Parameter-distribution consistency.** The value E=bP is uniformly distributed over $G_{1}\setminus \left\{0_{G_{1}}\right\}$, as specified in the real Setup. For every j∈{0,…,ℓ}, since $\beta_{j}$ and $\eta_{j}$ are independently uniform in $Z_{q}$ and *P* generates $G_{1}$, the values

$$T_{j}=\alpha_{j}E+\beta_{j}P,\;S_{j}=\gamma_{j}E+\eta_{j}P$$

are independently uniform in $G_{1}$, including $0_{G_{1}}$, as in the real Setup. Moreover, the target public key aP is uniformly distributed over $G_{1}\setminus \left\{0_{G_{1}}\right\}$, exactly as an ordinary public key rP for $r\leftarrow Z_{q}^{*}$.

For an encoded input *z*, let

$$\left(z_{1},\ldots ,z_{\ell }\right)={\hat{H}}_{2}\left(z\right),\;\left(z_{1}^{\prime },\ldots ,z_{\ell }^{\prime }\right)={\hat{H}}_{3}\left(z\right).$$

The public hash functions are

$$H_{2}\left(z\right)=T_{0}+\sum_{j=1}^{\ell }z_{j}T_{j},\;H_{3}\left(z\right)=S_{0}+\sum_{j=1}^{\ell }z_{j}^{\prime }S_{j}.$$

Using its stored coefficients, C can compute

$$\begin{array}{cccc} J_{2}\left(z\right) & =\alpha_{0}+\sum_{j=1}^{\ell }z_{j}\alpha_{j}, & B_{2}\left(z\right) & =\beta_{0}+\sum_{j=1}^{\ell }z_{j}\beta_{j},\\ J_{3}\left(z\right) & =\gamma_{0}+\sum_{j=1}^{\ell }z_{j}^{\prime }\gamma_{j}, & B_{3}\left(z\right) & =\eta_{0}+\sum_{j=1}^{\ell }z_{j}^{\prime }\eta_{j}, \end{array}$$

so that

$$H_{2}\left(z\right)=J_{2}\left(z\right)E+B_{2}\left(z\right)P,\;H_{3}\left(z\right)=J_{3}\left(z\right)E+B_{3}\left(z\right)P.$$

The coefficients $J_{2},J_{3}$ are defined over the integers; group scalar operations are performed modulo *q*.

*(3) Distributes parameters.*

The reduction sends

$$params=(G_{1},G_{2},q,e,P,E,P_{pub},{\left\{T_{j}\right\}}_{j=0}^{\ell },{\left\{S_{j}\right\}}_{j=0}^{\ell },H_{1},{\hat{H}}_{2},{\hat{H}}_{3})$$

to A, including the public hash indices and descriptions. It retains mk and the decomposition coefficients privately.

**Query phase.** The reduction C maintains the lists defined in Game 1. In addition to the lists defined in Game 1, C maintains a reduction-only list $L_{Sim}$ for proxy signing keys that are represented implicitly during signing-query simulations. Each entry records the matching delegation, identities, public keys, the ordered list *L*, and the random scalar $u_{PS_{i}}$ and public value $U_{PS_{i}}$. The corresponding proxy signing key is not stored in $L_{Sim}$ because C cannot compute it explicitly. Entries in $L_{Sim}$ do not represent key disclosure and are not entries in $L_{Exp}$. Let $q_{PU}$ be a polynomial upper bound on the number of distinct identities registered during the interaction, including registrations triggered by queries other than $Q_{\mathrm{PubK}}$. Before answering queries, C uniformly guesses one of these registration positions. When the identity at the guessed registration position is registered, C sets its public key to aP. Since *a* is uniform in $Z_{q}^{*}$, this public key is distributed exactly as rP for $r\leftarrow Z_{q}^{*}$ in the real key-generation algorithm. The identity registered at that position is denoted by $ID^{⋄}$. This is an internal guess by the reduction and does not require A to select its target identity in advance.

$Q_{\mathrm{PubK}}\left(ID_{i}\right)$: If $ID_{i}$ is already registered, C returns its current public key from $L_{Pub}$. Otherwise, it registers $ID_{i}$ as follows.

(1) If this is the guessed registration position, C sets

$$ID^{⋄}=ID_{i},\;R_{i}=aP.$$

It stores $\left(ID_{i},R_{i},⊥\right)$ in $L_{Key}$ and $\left(ID_{i},R_{i}\right)$ in $L_{Pub}$, where ⊥ indicates that the corresponding private key is unknown to C.

(2) Otherwise, C uniformly selects $r_{i}\in Z_{q}^{*}$ and computes

$$R_{i}=r_{i}P,\;h_{i}=H_{1}\left(ID_{i},R_{i}\right),\;d_{i}=\left(r_{i}+h_{i}x\right)\;\mathrm{mod}\;q.$$

It stores $\left(ID_{i},R_{i},d_{i}\right)$ in $L_{Key}$ and $\left(ID_{i},R_{i}\right)$ in $L_{Pub}$.

In both cases, C returns $R_{i}$. The same registration procedure is used whenever another query first introduces an unregistered identity. Internal key generation does not add an entry to $L_{Pri}$. Since *a* is uniformly distributed over $Z_{q}^{*}$, the initial public key aP has the same distribution as an honestly generated public key $r_{i}P$.

$Q_{\mathrm{PriK}}\left(ID_{i}\right)$: If $ID_{i}$ is unregistered, C first registers it using the procedure described in $Q_{\mathrm{PubK}}$. It then proceeds as follows.

(1) If $ID_{i}=ID^{⋄}$, C aborts the simulation, since the corresponding private key is unknown.

(2) Otherwise, C retrieves $\left(ID_{i},R_{i},d_{i}\right)$ from $L_{Key}$ and returns $d_{i}$ to A. It adds $ID_{i}$ to $L_{Pri}$ if it is not already recorded. Repeated queries return the same private key.

$Q_{\mathrm{Dele}}\left(w,ID_{OS},PK_{OS},L\right)$: The reduction C registers any unregistered identities using the procedure described in $Q_{\mathrm{PubK}}$. It checks the supplied public keys and the designated proxy group as specified in Game 1. If any check fails, it returns ⊥.

It computes

$$h_{OS}=H_{1}\left(ID_{OS},R_{OS}\right),$$

and proceeds as follows.

(1) If $ID_{OS}\neq ID^{⋄}$, C retrieves the corresponding private key $d_{OS}$, computes

$$K_{OS}=H_{2}\left(w,ID_{OS},R_{OS},L\right),$$

samples $u_{OS}$ uniformly from $Z_{q}^{*}$, and computes

$$U_{OS}=u_{OS}P,\;V_{OS}=d_{OS}E+u_{OS}K_{OS}.$$

(2) If $ID_{OS}=ID^{⋄}$, then $R_{OS}=aP$. C computes

$$K_{OS}=H_{2}\left(w,ID_{OS},R_{OS},L\right),$$

and, using its stored hash-generation coefficients, computes the integer coefficient *J* and a scalar $B\in Z_{q}$ such that

$$K_{OS}=JE+BP.$$

If J=0, it aborts the simulation. Otherwise, it samples *t* uniformly from $Z_{q}$ and computes

$$U_{OS}=tP-J^{-1}\left(aP\right).$$

If $U_{OS}=0_{G_{1}}$, it resamples *t* and recomputes $U_{OS}$ until $U_{OS}\neq 0_{G_{1}}$. It then computes

$$V_{OS}=h_{OS}xE+tK_{OS}-BJ^{-1}\left(aP\right).$$

In either case, C returns $\left(w,U_{OS},V_{OS}\right)$ together with *L* and stores

$$\left(w,ID_{OS},PK_{OS},L,\left(w,U_{OS},V_{OS}\right)\right)$$

in $L_{Dele}$. No entry is added to $L_{Pri}$.

$Q_{\mathrm{ProK}}$: The reduction C registers any unregistered identities as described in $Q_{\mathrm{PubK}}$. It verifies the delegation and checks the supplied public keys and the membership of $PS_{i}$ according to Game 1. If any check fails, it returns ⊥.

A matching entry is determined by the delegation, the original signer, the proxy signer, their public keys, and the ordered list *L*.

If a matching entry exists in $L_{\mathrm{Pro}}$, C retrieves the stored $\left(PSK_{PS_{i}},U_{PS_{i}}\right)$. It records the corresponding exposure tuple in $L_{\mathrm{Exp}}$ if it is not already present, and returns $\left(PSK_{PS_{i}},U_{PS_{i}}\right)$ to A.

If no matching entry exists in $L_{\mathrm{Pro}}$ but a matching entry exists in $L_{\mathrm{Sim}}$, the corresponding proxy signing key is represented implicitly and cannot be returned to A. In this case, C aborts this query simulation. It does not add an entry to $L_{\mathrm{Exp}}$.

If no matching entry exists in either list, C computes

$$K_{OS}=H_{2}\left(w,ID_{OS},R_{OS},L\right),\;h_{PS_{i}}=H_{1}\left(ID_{PS_{i}},R_{PS_{i}}\right),$$

and proceeds as follows.

(1) If $ID_{PS_{i}}\neq ID^{⋄}$, C retrieves the corresponding private key $d_{PS_{i}}$ from $L_{\mathrm{Key}}$, uniformly selects $u_{PS_{i}}\in Z_{q}^{*}$, and computes

$$U_{PS_{i}}=u_{PS_{i}}P,\;PSK_{PS_{i}}=u_{PS_{i}}K_{OS}+d_{PS_{i}}E+V_{OS}.$$

(2) If $ID_{PS_{i}}=ID^{⋄}$ and $R_{PS_{i}}=aP$, C uses its stored hash-generation coefficients to compute the integer coefficient *J* and a scalar $B\in Z_{q}$ such that

$$K_{OS}=JE+BP.$$

If J=0, C aborts this query simulation. Otherwise, it uniformly selects $t\in Z_{q}$ and computes

$$U_{PS_{i}}=tP-J^{-1}\left(aP\right).$$

If $U_{PS_{i}}=0_{G_{1}}$, it resamples *t* and recomputes $U_{PS_{i}}$ until $U_{PS_{i}}\neq 0_{G_{1}}$. It then computes

$$PSK_{PS_{i}}=h_{PS_{i}}xE+tK_{OS}-BJ^{-1}\left(aP\right)+V_{OS}.$$

This computation is consistent with ProK-Generate. For the implicit scalar $u_{PS_{i}}=t-aJ^{-1}$,

$$\begin{array}{cc} u_{PS_{i}}K_{OS}+d_{PS_{i}}E+V_{OS} & =\left(t-aJ^{-1}\right)\left(JE+BP\right)+\left(a+h_{PS_{i}}x\right)E+V_{OS}\\ & =tK_{OS}-BJ^{-1}\left(aP\right)+h_{PS_{i}}xE+V_{OS}. \end{array}$$

If $ID_{PS_{i}}=ID^{⋄}$ but $R_{PS_{i}}\neq aP$, C aborts this query simulation.

For either newly generated explicit key, C stores

$$\left(\left(w,U_{OS},V_{OS}\right),ID_{OS},PK_{OS},ID_{PS_{i}},PK_{PS_{i}},L,PSK_{PS_{i}},U_{PS_{i}}\right)$$

in $L_{\mathrm{Pro}}$. It records the corresponding exposure tuple in $L_{\mathrm{Exp}}$ and returns $\left(PSK_{PS_{i}},U_{PS_{i}}\right)$ to A. Internal generation of a key does not add an entry to $L_{\mathrm{Pri}}$.

$Q_{\mathrm{MPS}}\left(m,\left(w,U_{OS},V_{OS}\right),ID_{OS},PK_{OS},{\left\{ID_{PS_{i}}\right\}}_{i=1}^{n},{\left\{PK_{PS_{i}}\right\}}_{i=1}^{n}\right)$:

The reduction C verifies the delegation, checks that *m* complies with *w*, and checks the supplied public keys and designated proxy group according to Game 1. If any check fails, it returns ⊥. Otherwise, it proceeds as follows.

*(1) Partial signature generation.*

For each honest proxy signer $PS_{i}$, C searches $L_{\mathrm{Pro}}$ and $L_{\mathrm{Sim}}$ for a matching proxy signing-key record. A matching record has the same delegation, original signer, proxy signer, public keys, and ordered list *L*.

If a matching explicit key $\left(PSK_{PS_{i}},U_{PS_{i}}\right)$ exists in $L_{\mathrm{Pro}}$, C retrieves and uses it.

If a matching implicit-key record exists in $L_{\mathrm{Sim}}$, C retrieves $\left(u_{PS_{i}},U_{PS_{i}}\right)$. The corresponding proxy signing key is represented as

$$PSK_{PS_{i}}=u_{PS_{i}}K_{OS}+d_{PS_{i}}E+V_{OS},$$

but is not computed explicitly.

If no matching record exists and $ID_{PS_{i}}\neq ID^{⋄}$, C samples $u_{PS_{i}}\in Z_{q}^{*}$ and computes

$$U_{PS_{i}}=u_{PS_{i}}P,\;PSK_{PS_{i}}=u_{PS_{i}}K_{OS}+d_{PS_{i}}E+V_{OS}.$$

It stores this explicit key and its public value in $L_{\mathrm{Pro}}$. This internal generation does not add an entry to $L_{\mathrm{Exp}}$.

If no matching record exists and $ID_{PS_{i}}=ID^{⋄}$, C computes

$$z_{2}=\left(w,ID_{OS},R_{OS},L\right),\;K_{OS}=H_{2}\left(z_{2}\right)=J_{2}\left(z_{2}\right)E+B_{2}\left(z_{2}\right)P.$$

If $J_{2}\left(z_{2}\right)\neq 0$, C uses the internal explicit-key simulation described in $Q_{\mathrm{ProK}}$. It stores the resulting key in $L_{\mathrm{Pro}}$ without recording an exposure in $L_{\mathrm{Exp}}$.

If $J_{2}\left(z_{2}\right)=0$, C samples $u_{PS_{i}}\in Z_{q}^{*}$ and computes

$$U_{PS_{i}}=u_{PS_{i}}P.$$

It stores the matching delegation, identities, public keys, ordered list *L*, and the pair $\left(u_{PS_{i}},U_{PS_{i}}\right)$ in $L_{\mathrm{Sim}}$. It does not compute $PSK_{PS_{i}}$ explicitly.

If any required internal key-generation simulation aborts, C aborts this signing-query simulation.

When A controls some participants, it supplies their protocol messages as specified in Game 1. Using the public values from the signing interaction, C computes

$$U_{MPS}=\sum_{i=1}^{n}U_{PS_{i}},\;K_{PS}=H_{3}\left(m,w,ID_{OS},R_{OS},U_{MPS}\right).$$

For each honest signer with an explicit proxy signing key, C samples $x_{PS_{i}}\in Z_{q}^{*}$ and computes

$$X_{PS_{i}}=x_{PS_{i}}P,\;V_{PS_{i}}=x_{PS_{i}}K_{PS}+PSK_{PS_{i}}.$$

For an honest signer whose proxy signing key is represented implicitly in $L_{\mathrm{Sim}}$, let

$$z_{3}=\left(m,w,ID_{OS},R_{OS},U_{MPS}\right),\;K_{PS}=H_{3}\left(z_{3}\right)=J_{3}\left(z_{3}\right)E+B_{3}\left(z_{3}\right)P.$$

If $J_{3}\left(z_{3}\right)=0$, C aborts this signing-query simulation. Otherwise, it samples $t\in Z_{q}$ and computes

$$X_{PS_{i}}=tP-J_{3}{\left(z_{3}\right)}^{-1}\left(aP\right).$$

If $X_{PS_{i}}=0_{G_{1}}$, it resamples *t* until $X_{PS_{i}}\neq 0_{G_{1}}$. It then computes

$$V_{PS_{i}}=tK_{PS}-B_{3}\left(z_{3}\right)J_{3}{\left(z_{3}\right)}^{-1}\left(aP\right)+u_{PS_{i}}K_{OS}+h_{PS_{i}}xE+V_{OS},$$

where

$$h_{PS_{i}}=H_{1}\left(ID_{PS_{i}},R_{PS_{i}}\right).$$

This partial signature is consistent with MP-Sign. For the implicit signing scalar

$$x_{PS_{i}}=t-aJ_{3}{\left(z_{3}\right)}^{-1},$$

and the implicitly represented key

$$PSK_{PS_{i}}=u_{PS_{i}}K_{OS}+\left(a+h_{PS_{i}}x\right)E+V_{OS},$$

we have

$$\begin{array}{cc} x_{PS_{i}}K_{PS}+PSK_{PS_{i}}= & \left(t-aJ_{3}{\left(z_{3}\right)}^{-1}\right)\left(J_{3}\left(z_{3}\right)E+B_{3}\left(z_{3}\right)P\right)\\ & +u_{PS_{i}}K_{OS}+\left(a+h_{PS_{i}}x\right)E+V_{OS}\\ = & tK_{PS}-B_{3}\left(z_{3}\right)J_{3}{\left(z_{3}\right)}^{-1}\left(aP\right)\\ & +u_{PS_{i}}K_{OS}+h_{PS_{i}}xE+V_{OS}. \end{array}$$

The resampling condition ensures that $x_{PS_{i}}\in Z_{q}^{*}$. For each signing query, C uses fresh signing randomness and reuses the matching cached key state.

For each honest signer, C forms

$$\sigma_{PS_{i}}=\left(w,U_{OS},V_{OS},X_{PS_{i}},V_{PS_{i}}\right)$$

and records

$$\left(m,\left(w,U_{OS},V_{OS}\right),ID_{OS},PK_{OS},ID_{PS_{i}},PK_{PS_{i}},L,\sigma_{PS_{i}}\right)$$

in $L_{\mathrm{Sign}}$.

C returns the prescribed public messages and partial signatures to A. The records of released honest partial signatures are retained even if aggregation is not completed.

*(2) Verification and aggregation.*

When all *n* partial signatures are available, C verifies them according to MP-Sign. If any verification fails, it returns ⊥. Otherwise, it computes

$$V_{MPS}=\sum_{i=1}^{n}V_{PS_{i}},\;X_{MPS}=\sum_{i=1}^{n}X_{PS_{i}},$$

and returns

$$\sigma_{MPS}=\left(w,U_{OS},U_{MPS},X_{MPS},V_{MPS}\right).$$

It stores

$$\left(m,\left(w,U_{OS},V_{OS}\right),ID_{OS},PK_{OS},{\left\{ID_{PS_{i}}\right\}}_{i=1}^{n},{\left\{PK_{PS_{i}}\right\}}_{i=1}^{n},L,\sigma_{MPS}\right)$$

in $L_{\mathrm{Sign}}$.

Internal proxy-key generation does not constitute key disclosure and does not add an entry to $L_{\mathrm{Exp}}$.

**Lemma** **1 (Coupling of the query simulation).** *The real experiment and the reduction’s simulation can be coupled so that their adversary-visible transcripts agree until the first simulation abort.*

**Proof.**  Uniform masking in $T_{j}=\alpha_{j}E+\beta_{j}P$ and $S_{j}=\gamma_{j}E+\eta_{j}P$ makes the public parameters independent of the hidden coefficient vectors. For analysis, generate a complete real execution T using independent real-game randomness, and sample the hidden coefficients independently of T; the corresponding masking scalars give an equivalent representation of the parameter generation.

Couple simulated randomness to real randomness by t=u+a/J for delegation and explicit-key simulation, and $t=x_{\mathrm{sig}}+a/J_{3}$ for implicit-key signing, where *u* denotes the corresponding real key-generation or delegation randomness and $x_{\mathrm{sig}}$ denotes the real signing randomness $x_{PS_{i}}$. For the probability analysis, the target and query bit strings are read from the independently generated real execution T. Whenever the denominators are nonzero, these translations preserve the required distributions and produce identical responses. Both executions reuse matching cached keys. On the sufficient coefficient event, any target proxy key later exposed has nonzero $J_{2}$ and is therefore generated explicitly from its first use.

Identical visible histories induce identical subsequent adaptive queries. Thus the executions agree without abort on this event, while the bit strings determined by T remain independent of the hidden coefficients. □

**Lemma** **2 (Coefficient event bound).** *Let Q≥1, M=2Q, and sample $c_{0},\ldots ,c_{\ell }$ uniformly and independently from {0,…,M−1}, and k uniformly and independently from {0,…,ℓ}. Define*

$$J\left(v\right)=c_{0}+\sum_{j=1}^{\ell }v_{j}c_{j}-Mk.$$

For fixed bit strings $v^{*},v^{\left(1\right)},\ldots ,v^{\left(N\right)}\in {\left\{0,1\right\}}^{\ell }$, independent of these samples, with 0≤N≤Q and $v^{\left(i\right)}\neq v^{*}$ for every i,

$$\mathrm{Pr}\;\left[J\left(v^{*}\right)=0,\;J\left(v^{\left(i\right)}\right)\neq 0\;\forall i\right]\geq \frac{1}{4Q(\ell +1)}.$$

**Proof.**  Write $S\left(v\right)=c_{0}+\sum_{j}v_{j}c_{j}$ and F(v)=S(v)modM. The uniform choice of $c_{0}$ gives $\mathrm{Pr}[F\left(v^{*}\right)=0]=1/M$. For each *i*, the strings differ at some coordinate *h*. Conditional on the remaining coefficients, the map from $\left(c_{0},c_{h}\right)$ to $\left(F\left(v^{*}\right),F\left(v^{\left(i\right)}\right)\right)$ has determinant $v_{h}^{\left(i\right)}-v_{h}^{*}=\pm 1$, and is therefore invertible modulo *M*. Hence,

$$\mathrm{Pr}\;\left[F\left(v^{\left(i\right)}\right)=0|F\left(v^{*}\right)=0\right]=\frac{1}{M}.$$

By the union bound, all query residues are nonzero with conditional probability at least 1−N/M.

When $F(v^{*})=0$, the bound $0\leq S\left(v^{*}\right)<M\left(\ell +1\right)$ ensures a unique $k=S\left(v^{*}\right)/M\in \left\{0,\ldots ,\ell \right\}$, selected with probability 1/(ℓ+1). Nonzero query residues imply nonzero integer coefficients. Therefore,

$$\begin{array}{cc} & \mathrm{Pr}\;\left[J\left(v^{*}\right)=0,\;J\left(v^{\left(i\right)}\right)\neq 0\;\forall i\right]\\ & \;\geq \frac{1-N/M}{M(\ell +1)}\geq \frac{1}{4Q(\ell +1)}. \end{array}$$

Moreover, under the parameter condition q>M(ℓ+1), |J(v)|<M(ℓ+1)<q, so integer nonzero coefficients remain nonzero modulo *q*. □

**Forgery Phase.** The adversary A outputs either a multi-proxy signature

$$\sigma_{MPS}^{*}=\left(w^{*},U_{OS}^{*},U_{MPS}^{*},X_{MPS}^{*},V_{MPS}^{*}\right)$$

or a delegation $\left(w^{*},U_{OS}^{*},V_{OS}^{*}\right)$, together with the identities and public keys required by Game 1.

**Scenario 1: Delegation forgery.**

Suppose that $\left(w^{*},U_{OS}^{*},V_{OS}^{*}\right)$ satisfies the winning conditions of Scenario 1 in Game 1.

If $ID_{OS}^{*}\neq ID^{⋄}$, or if the public key used to verify the forgery is not $R_{OS}^{*}=aP$, C aborts. Otherwise, it computes

$$h_{OS}^{*}=H_{1}\left(ID_{OS}^{*},R_{OS}^{*}\right),\;K_{OS}^{*}=H_{2}\left(w^{*},ID_{OS}^{*},R_{OS}^{*},L^{*}\right).$$

Using its stored hash-generation coefficients, C obtains $J_{2}^{*}\in Z$ and $B_{2}^{*}\in Z_{q}$ such that

$$K_{OS}^{*}=J_{2}^{*}E+B_{2}^{*}P.$$

If $J_{2}^{*}\neq 0$, C aborts. Otherwise, $K_{OS}^{*}=B_{2}^{*}P$.

Since the final delegation passes verification,

$$\begin{array}{cc} e(V_{OS}^{*},P) & =e\left(E,aP+h_{OS}^{*}xP\right)e\left(B_{2}^{*}P,U_{OS}^{*}\right)\\ & =e(abP+h_{OS}^{*}xE+B_{2}^{*}U_{OS}^{*},P). \end{array}$$

By non-degeneracy and the prime order of $G_{1}$, the map V↦e(V,P) is injective. Therefore,

$$V_{OS}^{*}=abP+h_{OS}^{*}xE+B_{2}^{*}U_{OS}^{*}.$$

The reduction outputs

$$abP=V_{OS}^{*}-h_{OS}^{*}xE-B_{2}^{*}U_{OS}^{*}.$$

**Probability Analysis for Scenario 1.**

Let $q_{PU}$ be a polynomial upper bound on the number of distinct identity registrations. Let $Q_{1}\geq 1$ be a fixed polynomial upper bound on the number of distinct $H_{2}$ inputs for which the simulation requires a nonzero coefficient $J_{2}$. These inputs include the target identity’s delegation queries and any other simulation queries for which $J_{2}\neq 0$ is required. Set $M_{1}=2Q_{1}$.

Let $\varepsilon_{1}^{\mathrm{good}}$ denote the probability that A produces a successful Scenario 1 forgery with an unchanged target public key, with all required non-target private keys available to C, and with no collision under ${\hat{H}}_{2}$ between the target input and a distinct input requiring a nonzero $J_{2}$.

Define the events:

$$\begin{array}{cc} \pi_{1} & :\mathrm{such}\;a\;\mathrm{successful}\;\mathrm{Scenario}\;1\;\mathrm{forgery}\;\mathrm{occurs},\\ \pi_{2} & :C\;\mathrm{correctly}\;\mathrm{guesses}\;\mathrm{the}\;\mathrm{target}\;\mathrm{identity}’s\;\mathrm{registration}\;\mathrm{position},\\ \pi_{3} & :\mathrm{the}\;\mathrm{query}\;\mathrm{simulation}\;\mathrm{completes}\;\mathrm{and}\;J_{2}^{*}=0. \end{array}$$

Then

$$\mathrm{Pr}\left[\pi_{1}\right]=\varepsilon_{1}^{\mathrm{good}},\;\mathrm{Pr}\left[\pi_{2}|\pi_{1}\right]=\frac{1}{q_{PU}}.$$

For a fixed eligible real-game execution, let $v^{*}$ be the target bit string output by ${\hat{H}}_{2}$, and let $v^{\left(1\right)},\ldots ,v^{\left(N\right)}$ be the distinct query bit strings requiring nonzero $J_{2}$, where $N\leq Q_{1}$. Eligibility ensures that $v^{\left(i\right)}\neq v^{*}$ for every *i*. Applying the Coefficient event bound lemma with $Q=Q_{1}$ and $M=M_{1}$ gives

$$\begin{array}{cc} \mathrm{Pr}[\pi_{3}|\pi_{1}\wedge \pi_{2}] & \geq \frac{1}{M_{1}}\left(1-\frac{N}{M_{1}}\right)\frac{1}{\ell +1}\\ & \geq \frac{1}{4Q_{1}\left(\ell +1\right)}. \end{array}$$

By the coupling lemma, the complete real execution T is independent of the hidden coefficient vector. Conditional on any eligible T, the target and query bit strings are fixed, so the Coefficient event bound lemma applies. Averaging this pointwise lower bound over eligible real executions gives the same bound for adaptive queries.

Whenever $\pi_{1},\pi_{2},\pi_{3}$ occur, the extraction in Scenario 1 outputs the correct CDH value. Hence,

$$\begin{array}{cc} \mathrm{Pr}[C\;\mathrm{solves}\;\mathrm{CDH}] & \geq \mathrm{Pr}\left[\pi_{1}\right]\mathrm{Pr}\left[\pi_{2}|\pi_{1}\right]\mathrm{Pr}\left[\pi_{3}|\pi_{1}\wedge \pi_{2}\right]\\ & \geq \frac{\varepsilon_{1}^{\mathrm{good}}}{4q_{PU}Q_{1}\left(\ell +1\right)}. \end{array}$$

By the collision resistance of ${\hat{H}}_{2}$ and the polynomial number of queries, the probability that the target input collides with a distinct, relevant query input is negligible. Hence, $\varepsilon_{1}^{\mathrm{good}}$ differs from the Scenario 1 winning probability by at most a negligible amount.

**Scenario 2: CDH extraction.**

Suppose that A outputs a signature

$$\sigma_{MPS}^{*}=\left(w^{*},U_{OS}^{*},U_{MPS}^{*},X_{MPS}^{*},V_{MPS}^{*}\right)$$

on $m^{*}$ satisfying the winning conditions of Scenario 2 in Game 1. Let $PS_{1}$ denote the honest proxy signer.

If $ID_{PS_{1}}^{*}\neq ID^{⋄}$, or if the public key used in the forgery is not $R_{PS_{1}}^{*}=aP$, C aborts. Otherwise,

$$h_{PS_{1}}^{*}=H_{1}\left(ID_{PS_{1}}^{*},R_{PS_{1}}^{*}\right),\;d_{PS_{1}}^{*}=\left(a+h_{PS_{1}}^{*}x\right)\;\mathrm{mod}\;q.$$

The reduction evaluates

$$\begin{array}{cc} K_{OS}^{*} & =H_{2}\left(w^{*},ID_{OS}^{*},R_{OS}^{*},L^{*}\right),\\ K_{PS}^{*} & =H_{3}\left(m^{*},w^{*},ID_{OS}^{*},R_{OS}^{*},U_{MPS}^{*}\right). \end{array}$$

Using the coefficients retained during its parameter generation, C computes

$$K_{OS}^{*}=J_{2}^{*}E+B_{2}^{*}P,\;K_{PS}^{*}=J_{3}^{*}E+B_{3}^{*}P.$$

The extraction requires

$$J_{2}^{*}=0,\;J_{3}^{*}=0,$$

and knowledge of $d_{OS}^{*},d_{PS_{2}}^{*},\ldots ,d_{PS_{n}}^{*}$ for the corresponding public keys in the forgery. These private keys are available from $L_{\mathrm{Key}}$ for the corrupted participants in Scenario 2. If any extraction condition fails, C does not output a CDH solution in this execution.

Since MP-Verify accepts the final forgery, its verification equation, bilinearity, and non-degeneracy imply

$$V_{MPS}^{*}=\left(nd_{OS}^{*}+\sum_{i=1}^{n}d_{PS_{i}}^{*}\right)E+B_{2}^{*}\left(nU_{OS}^{*}+U_{MPS}^{*}\right)+B_{3}^{*}X_{MPS}^{*}.$$

Substituting $d_{PS_{1}}^{*}=\left(a+h_{PS_{1}}^{*}x\right)\;\mathrm{mod}\;q$ gives

$$\begin{array}{cc} V_{MPS}^{*}= & abP+\left(nd_{OS}^{*}+h_{PS_{1}}^{*}x+\sum_{i=2}^{n}d_{PS_{i}}^{*}\right)E\\ & +B_{2}^{*}\left(nU_{OS}^{*}+U_{MPS}^{*}\right)+B_{3}^{*}X_{MPS}^{*}. \end{array}$$

Therefore, C outputs

$$\begin{array}{cc} abP= & V_{MPS}^{*}-B_{2}^{*}\left(nU_{OS}^{*}+U_{MPS}^{*}\right)-B_{3}^{*}X_{MPS}^{*}\\ & -\left(nd_{OS}^{*}+h_{PS_{1}}^{*}x+\sum_{i=2}^{n}d_{PS_{i}}^{*}\right)E. \end{array}$$

All terms on the right-hand side are known to C under the stated conditions. The reduction performs this extraction only from the adversary’s final forgery.

**Probability Analysis for Scenario 2.**

Let $q_{PU}$ be a polynomial upper bound on the number of distinct identity registrations. Let $Q_{1},Q_{2}\geq 1$ be fixed polynomial upper bounds on the numbers of distinct query bit strings that impose nonzero-coefficient conditions for $H_{2}$ and $H_{3}$, respectively. Set

$$M_{1}=2Q_{1},\;M_{2}=2Q_{2}.$$

For a fixed real execution T and the guessed target identity, let $Z_{2}\left(T\right)$ contain the distinct $H_{2}$ inputs of valid delegation queries in which the target is the original signer, and valid proxy-key exposure queries in which the target is the proxy signer. Its size is bounded by $Q_{1}$. Requiring nonzero $J_{2}$ on this set ensures that a key later exposed is generated explicitly from its first use.

Let $Z_{3}\left(T\right)$ contain all distinct $H_{3}$ inputs at which the target proxy releases a partial signature, including queries answered using explicit keys. Its size is bounded by $Q_{2}$. This conservative set covers every implicit-key signing query. Both sets are determined by T and the target identity, independently of the hidden coefficients.

Let $\varepsilon_{2}^{\mathrm{good}}$ be the probability that A produces a successful Scenario 2 forgery with an unchanged target public key, with the other private keys required for extraction are available, and with no collision under either ${\hat{H}}_{2}$ or ${\hat{H}}_{3}$ between distinct relevant inputs. The relevant inputs include the target inputs and all query inputs are counted in $Q_{1}$ and $Q_{2}$.

Define the events:

$$\begin{array}{cc} \pi_{1} & :\mathrm{such}\;a\;\mathrm{successful}\;\mathrm{Scenario}\;2\;\mathrm{forgery}\;\mathrm{occurs},\\ \pi_{2} & :C\;\mathrm{correctly}\;\mathrm{guesses}\;\mathrm{the}\;\mathrm{honest}\;\mathrm{proxy}’s\;\mathrm{registration}\;\mathrm{position},\\ \pi_{3} & :\mathrm{the}\;H_{2}-\mathrm{coefficient}\;\mathrm{conditions}\;\mathrm{hold}\;\mathrm{and}\;\mathrm{all}\;H_{2}-\mathrm{dependent}\;\mathrm{queries}\;\mathrm{are}\;\mathrm{answered},\\ \pi_{4} & :J_{3}^{*}=0\;\mathrm{and}\;\mathrm{all}\;H_{3}-\mathrm{dependent}\;\mathrm{signing}\;\mathrm{queries}\;\mathrm{are}\;\mathrm{answered}. \end{array}$$

Here, $\pi_{3}$ requires $J_{2}^{*}=0$ and requires $J_{2}\neq 0$ for every distinct $H_{2}$ query input counted in $Q_{1}$. Event $\pi_{4}$ requires $J_{3}^{*}=0$ and requires $J_{3}\neq 0$ for every distinct $H_{3}$ query input counted in $Q_{2}$.

Fix an eligible real execution T and a correct target-identity guess. By the coupling lemma, the target bit strings and the sets $Z_{2}\left(T\right)$ and $Z_{3}\left(T\right)$ are fixed independently of the hidden coefficients. Freshness and collision exclusion ensure that each target bit string differs from the query bit strings in its corresponding set.

Applying the Coefficient event bound lemma to the two independently sampled coefficient families gives a lower bound of $1/[16Q_{1}Q_{2}{\left(\ell +1\right)}^{2}]$ for the joint coefficient event. On this event, the coupled simulation completes and extraction succeeds. Averaging over eligible real executions therefore yields

$$\mathrm{Pr}\left[\pi_{3}\wedge \pi_{4}|\pi_{1}\wedge \pi_{2}\right]\geq \frac{1}{16Q_{1}Q_{2}{\left(\ell +1\right)}^{2}}.$$

No independence between $\pi_{3}$ and $\pi_{4}$ is assumed.

The target identity’s registration position is guessed with probability at least $1/q_{PU}$. Therefore,

$$\begin{array}{cc} \mathrm{Pr}[C\;\mathrm{solves}\;\mathrm{CDH}] & \geq \mathrm{Pr}\left[\pi_{1}\right]\mathrm{Pr}\left[\pi_{2}|\pi_{1}\right]\;\mathrm{Pr}\left[\pi_{3}\wedge \pi_{4}|\pi_{1}\wedge \pi_{2}\right]\\ & \geq \frac{\varepsilon_{2}^{\mathrm{good}}}{16q_{PU}Q_{1}Q_{2}{\left(\ell +1\right)}^{2}}. \end{array}$$

The probability of a collision under either collision-resistant hash family is negligible for a polynomial number of queries. The freshness conditions in Scenario 2 exclude the target signing input from the corresponding prohibited signing queries. Consequently, the collision-related loss between the Scenario 2 winning probability and $\varepsilon_{2}^{\mathrm{good}}$ is negligible.

Since $q_{PU},Q_{1},Q_{2}$, and *ℓ* are polynomially bounded, a non-negligible Scenario 2 winning probability yields a non-negligible CDH success probability, up to negligible collision-related loss. This contradicts the CDH assumption. Hence, the scheme is existentially unforgeable under Scenario 2 of Game 1. The coupling and coefficient-event lemmas establish the required simulation and extraction probabilities; these are not additional assumptions.

**Scenario 3: CDH extraction.**

Suppose that A outputs a signature

$$\sigma_{MPS}^{*}=\left(w^{*},U_{OS}^{*},U_{MPS}^{*},X_{MPS}^{*},V_{MPS}^{*}\right)$$

on $m^{*}$ satisfying Scenario 3 of Game 1. The original signer remains honest, whereas all *n* proxy signers are compromised. Assume that 1≤n<q.

If $ID_{OS}^{*}\neq ID^{⋄}$, or if the original signer’s public key in the forgery is not aP, C aborts. Otherwise,

$$R_{OS}^{*}=aP,\;d_{OS}^{*}=\left(a+h_{OS}^{*}x\right)\;\mathrm{mod}\;q,$$

where

$$h_{OS}^{*}=H_{1}\left(ID_{OS}^{*},R_{OS}^{*}\right).$$

The reduction evaluates

$$\begin{array}{cc} K_{OS}^{*} & =H_{2}\left(w^{*},ID_{OS}^{*},R_{OS}^{*},L^{*}\right),\\ K_{PS}^{*} & =H_{3}\left(m^{*},w^{*},ID_{OS}^{*},R_{OS}^{*},U_{MPS}^{*}\right). \end{array}$$

Using the coefficients retained during its parameter generation, C computes

$$K_{OS}^{*}=J_{2}^{*}E+B_{2}^{*}P,\;K_{PS}^{*}=J_{3}^{*}E+B_{3}^{*}P.$$

The extraction requires

$$J_{2}^{*}=0,\;J_{3}^{*}=0,$$

and knowledge of the private keys $d_{PS_{1}}^{*},\ldots ,d_{PS_{n}}^{*}$ corresponding to the proxy public keys used in the forgery. Since all proxy signers are compromised in Scenario 3, these private keys are available to C from the key-generation and exposure records, provided their public keys are unchanged. If any required private key is unavailable, or either coefficient condition fails, C aborts this extraction.

Since MP-Verify accepts the forgery, the verification equation, bilinearity, and nondegeneracy imply

$$V_{MPS}^{*}=\left(nd_{OS}^{*}+\sum_{i=1}^{n}d_{PS_{i}}^{*}\right)E+B_{2}^{*}\left(nU_{OS}^{*}+U_{MPS}^{*}\right)+B_{3}^{*}X_{MPS}^{*}.$$

Substituting E=bP and $d_{OS}^{*}=\left(a+h_{OS}^{*}x\right)\;\mathrm{mod}\;q$ yields

$$\begin{array}{cc} V_{MPS}^{*}= & nabP+(nh_{OS}^{*}x+\sum_{i=1}^{n}d_{PS_{i}}^{*})E\\ & +B_{2}^{*}\left(nU_{OS}^{*}+U_{MPS}^{*}\right)+B_{3}^{*}X_{MPS}^{*}. \end{array}$$

Since 1≤n<q, the inverse $n^{-1}$ exists in $Z_{q}$. Therefore, C computes

$$\begin{array}{cc} abP=n^{-1}[ & V_{MPS}^{*}-B_{2}^{*}\left(nU_{OS}^{*}+U_{MPS}^{*}\right)-B_{3}^{*}X_{MPS}^{*}\\ & -(nh_{OS}^{*}x+\sum_{i=1}^{n}d_{PS_{i}}^{*})E]. \end{array}$$

Every term on the right-hand side is computable under the stated conditions.

**Probability Analysis for Scenario 3.**

Let $q_{PU}$ bound the number of distinct identity registrations, and let $Q_{1}\geq 1$ bound the number of distinct $H_{2}$ inputs for the target original signer that require a nonzero coefficient during simulation. The bound includes every target delegation query whose simulation requires $J_{2}\neq 0$. Set

$$M_{1}=2Q_{1},\;Q_{2}=1,\;M_{2}=2.$$

Let $\varepsilon_{3}^{\mathrm{good}}$ denote the probability of a successful Scenario 3 forgery for which the original signer’s public key is unchanged, all proxy private keys required for extraction are available, and the target $H_{2}$ bit string differs from every target $H_{2}$ query bit string requiring a nonzero coefficient.

Define:

$\pi_{1}$: such a successful forgery occurs;

$\pi_{2}$: C correctly guesses the original signer’s registration position;

$\pi_{3}$: all query simulations complete without aborting, including satisfaction of every required nonzero-coefficient condition;

$\pi_{4}$: $J_{2}^{*}=J_{3}^{*}=0$.

Under the distribution-preserving coupling, the chain rule gives

$$\begin{array}{cc} \mathrm{Pr}[C\;\mathrm{solves}\;\mathrm{CDH}]= & \mathrm{Pr}\left[\pi_{1}\right]\;\mathrm{Pr}\left[\pi_{2}|\pi_{1}\right]\;\mathrm{Pr}\left[\pi_{3}|\pi_{1}\wedge \pi_{2}\right]\\ & \cdot \mathrm{Pr}[\pi_{4}|\pi_{1}\wedge \pi_{2}\wedge \pi_{3}]. \end{array}$$

By definition and the hidden uniform guess,

$$\mathrm{Pr}\left[\pi_{1}\right]=\varepsilon_{3}^{\mathrm{good}},\;\mathrm{Pr}\left[\pi_{2}|\pi_{1}\right]=\frac{1}{q_{PU}}.$$

For a fixed eligible real-game execution, let $N_{1}\leq Q_{1}$ be the number of distinct target ${\hat{H}}_{2}$ query bit strings requiring nonzero $J_{2}$. By eligibility, each of these query bit strings differs from the target forgery bit string. Applying the Coefficient event bound lemma with $Q=Q_{1}$, $M=M_{1}$, and $N=N_{1}$ gives

$$\mathrm{Pr}\left[J_{2}^{*}=0,\;\mathrm{all}\;\mathrm{required}\;J_{2}\neq 0\right]\geq \frac{1}{4Q_{1}\left(\ell +1\right)}.$$

In Scenario 3, all proxy private keys are available to C. Thus, proxy-key generation and signing do not require a nonzero coefficient for $H_{3}$ in order to be simulated. The reduction evaluates $H_{3}$ directly. Accordingly, there are no $H_{3}$ query constraints to satisfy, and the target condition $J_{3}^{*}=0$ is the only $H_{3}$ coefficient condition. Applying the Coefficient event bound lemma with $Q=Q_{2}=1$, $M=M_{2}=2$, and N=0 gives

$$\mathrm{Pr}\left[J_{3}^{*}=0\right]\geq \frac{1}{4(\ell +1)}.$$

Fix an eligible real execution T and a correct target-identity guess. By the coupling lemma, the target and query bit strings are independent of the hidden coefficients. Applying the Coefficient event bound lemma to the two independent coefficient families gives a joint coefficient-event probability of at least $1/[16Q_{1}{\left(\ell +1\right)}^{2}]$. On this event, simulation and extraction succeed. Averaging over eligible real executions yields

$$\mathrm{Pr}\left[\pi_{3}\wedge \pi_{4}|\pi_{1}\wedge \pi_{2}\right]\geq \frac{1}{16Q_{1}{\left(\ell +1\right)}^{2}}.$$

No independence between $\pi_{3}$ and $\pi_{4}$ is required.

Consequently,

$$\mathrm{Pr}\left[C\;\mathrm{solves}\;\mathrm{CDH}\right]\geq \frac{\varepsilon_{3}^{\mathrm{good}}}{16q_{PU}Q_{1}{\left(\ell +1\right)}^{2}}.$$

By the collision resistance of ${\hat{H}}_{2}$ and the polynomial number of queries, the probability that the target input collides with a distinct relevant query input is negligible. Hence, $\varepsilon_{3}^{\mathrm{good}}$ differs from the Scenario 3 winning probability by at most a negligible amount.

If A wins Scenario 3 with non-negligible probability $\varepsilon_{3}^{\mathrm{good}}$, then C solves the CDH problem with non-negligible probability at least

$$\frac{\varepsilon_{3}^{\mathrm{good}}}{16q_{PU}Q_{1}{\left(\ell +1\right)}^{2}}.$$

Since $q_{PU}$, $Q_{1}$, and *ℓ* are polynomially bounded, this contradicts the CDH assumption. Therefore, the scheme is existentially unforgeable under Scenario 3 of Game 1 in the standard model.

By the union bound, the probability of winning Game 1 is at most $\varepsilon_{1}+\varepsilon_{2}+\varepsilon_{3}$. Since each term is negligible, the overall winning probability is negligible, completing the proof of Theorem 1.

### 6.2. Further Security Discussion

**Separate generation of partial proxy signing keys.**

The scheme includes a separate proxy signing key generation procedure. After verifying the delegation, each proxy signer computes

$$PSK_{PS_{i}}=u_{PS_{i}}K_{OS}+d_{PS_{i}}E+V_{OS}.$$

Subsequent signing uses $PSK_{PS_{i}}$ rather than directly accessing the long-term private key $d_{PS_{i}}$. This separates proxy-key generation from online signature generation.

**Long-term private-key protection under proxy-key exposure.**

We consider an honestly generated target public key that has not been replaced. The adversary may obtain the target user’s partial proxy signing keys and the associated public information, but not its long-term private scalar through a private-key extraction query. The objective is to recover that scalar.

Suppose that an adversary can recover $d_{PS_{i}}$ under these conditions. Given a CDH instance (P,aP,bP), a reduction uniformly selects $x,s\in Z_{q}^{*}$ and sets

$$P_{pub}=xP,\;E=sP,\;R_{PS_{i}}=aP.$$

All remaining parameters are generated as in the real scheme. The target key satisfies

$$h_{PS_{i}}=H_{1}\left(ID_{PS_{i}},R_{PS_{i}}\right),\;d_{PS_{i}}=a+h_{PS_{i}}x\;\left(\mathrm{mod}\;q\right).$$

Although the reduction does not know $d_{PS_{i}}$, it can compute

$$d_{PS_{i}}E=s\left(aP\right)+h_{PS_{i}}xE.$$

Consequently, for each valid delegation, the reduction selects $u_{PS_{i}}$ as in the real algorithm and computes

$$PSK_{PS_{i}}=u_{PS_{i}}K_{OS}+s\left(aP\right)+h_{PS_{i}}xE+V_{OS}.$$

The resulting partial proxy signing key has exactly the distribution prescribed by the scheme. The reduction can return this key and $U_{PS_{i}}=u_{PS_{i}}P$, and can generate partial signatures using the simulated proxy key. It can also disclose $u_{PS_{i}}$ if this is included in the exposure experiment.

If the adversary outputs the correct scalar $d_{PS_{i}}$, the reduction computes

$$a=d_{PS_{i}}-h_{PS_{i}}x\;\left(\mathrm{mod}\;q\right)$$

and returns

$$abP=\left(d_{PS_{i}}-h_{PS_{i}}x\right)\left(bP\right).$$

Thus, for a fixed target identity, successful private-scalar recovery yields a CDH solution with the same success probability.

**Partial multi-proxy signature verification.**

Before aggregation, the clerk verifies every partial signature using

$$\begin{array}{cc} e(V_{PS_{i}},P)= & e\;\left(E,R_{OS}+R_{PS_{i}}+\left(h_{OS}+h_{PS_{i}}\right)P_{pub}\right)\\ & \cdot e(X_{PS_{i}},K_{PS})\\ & \cdot e(K_{OS},U_{OS}+U_{PS_{i}}). \end{array}$$

The clerk aggregates the partial signatures only if all verification equations hold. Otherwise, it rejects the invalid partial signature and does not produce the aggregate signature from that collection. This provides an explicit check of each partial signature before aggregation. □

## 7. Performance Analysis

We compare the security-related features of representative identity-based multi-proxy signature schemes. For the computational-cost and representation-size comparisons, we additionally include a certificateless multi-proxy signature scheme as a reference.

Table 2 compares the security-related features and reported security analyses of the identity-based multi-proxy signature schemes. The certificateless multi-proxy signature scheme is excluded from this table because it uses a different key-generation framework and adversarial model, including public-key replacement and attacks by a malicious key-generation center. Its security guarantees are therefore not directly comparable under the same criteria. Nevertheless, it is included in the subsequent computational-cost and representation-size comparisons as a reference for collaborative proxy signing.

Table 2 distinguishes reported unforgeability proofs, partial proxy signing-key generation, resistance to long-term private-key recovery under PSK exposure, and verification of individual partial signatures. These are separate properties and should not be interpreted as interchangeable measures of security. The table records the proof models and principal assumptions reported for the compared constructions; it does not rank their overall security.

Li et al. [19] and Xu et al. [20] discuss individual security properties without providing formal reduction proofs. Cao et al. [21], Chen et al. [25], and Shin et al. [26] report CDH-based proofs in the ROM, whereas Gu et al. [24] present a construction and security analysis in the SM. A reported proof alone does not establish that a construction is secure. For example, Xiong et al. [22] showed that Cao et al.’s scheme permits an ordinary signature to be used to construct an unauthorized multi-proxy signature. Our unforgeability analysis is in the SM and relies on the CDH assumption and the collision resistance of ${\hat{H}}_{2}$ and ${\hat{H}}_{3}$. It provides a separate reduction for each of the three forgery scenarios in Game 1. Each reduction’s success probability accounts for its query simulation, extraction conditions, and negligible collision-related loss, as detailed in the preceding section.

The schemes of Li, Cao, Gu, and Shin include a proxy signing-key generation procedure [19,21,24,26]. In Xu et al.’s scheme [20], the original signer generates the partial proxy signing keys without using the proxies’ long-term private keys. Consequently, the recovery mechanism considered here, which extracts a proxy’s long-term private key from a PSK containing that key’s contribution, is not applicable. Chen et al.’s scheme [25] generates partial signatures directly from the long-term private key and delegation information, without a separate proxy signing-key generation procedure. In our scheme, each proxy first verifies the delegation and then derives an individual PSK for subsequent signing.

Separate proxy signing-key generation does not automatically prevent long-term private-key recovery. In the schemes of Li and Cao, the algebraic relationship between a PSK and the corresponding delegation allows recovery of the proxy’s long-term private key by removing the delegation contribution and, where necessary, inverting a known scalar [19,21]. Shin et al. [26] analyze these recovery mechanisms and report protection for Gu et al.’s construction and their own construction under the exposure settings they consider. These results must be interpreted with respect to the information available to the adversary. In particular, Shin et al.’s construction uses independent secret randomness in the PSK, and the direct recovery argument changes if that randomness is also disclosed.

In our scheme, the partial proxy signing key is

$$PSK_{PS_{i}}=u_{PS_{i}}K_{OS}+d_{PS_{i}}E+V_{OS}.$$

If the PSK, delegation, and random scalar $u_{PS_{i}}$ are exposed, the adversary can compute

$$PSK_{PS_{i}}-u_{PS_{i}}K_{OS}-V_{OS}=d_{PS_{i}}E.$$

This calculation reveals a group element rather than the private scalar $d_{PS_{i}}$. The preceding private-key recovery analysis shows how recovery of that scalar yields a CDH solution for an honestly generated target public key that has not been replaced. This property concerns protection of the long-term private scalar; it does not prevent signing with a compromised PSK.

The schemes of Li, Cao, Chen, and Shin support verification of individual partial multi-proxy signatures before aggregation [19,21,25,26]. Xu’s sequential construction and Gu’s construction do not provide the same per-contribution verification procedure [20,24]. In our scheme, the clerk verifies every partial signature and constructs the aggregate signature only if all checks succeed. This allows invalid contributions to be rejected before aggregation. By contrast, failure of a final aggregate verification alone does not identify which individual contribution is invalid.

The construction requires valid contributions from all designated proxy signers and does not provide threshold signing. If any designated UAV becomes unavailable, loses connectivity, or refuses to contribute, the joint signature cannot be completed until all required contributions are available. Partial signature verification detects invalid contributions but does not resolve missing contributions. Changing the signer group requires a new warrant and delegation binding the revised identities and ordered public-key list *L*. The following analysis therefore evaluates the specified cryptographic operations and representation sizes under explicit counting and encoding assumptions.

We next compare the computational cost of our scheme with those of existing schemes [19,21,23,24,25,26]. We use the primitive-operation timings reported in Table 1 of He et al. [27] as a common benchmark. These operations were measured using the MIRACL library on a Samsung Galaxy S5 smartphone with a quad-core processor, 2 GB RAM, and Android 4.4.2. The pairing benchmark uses a supersingular elliptic curve with a 160-bit subgroup order and a 512-bit underlying field parameter.

Let $W_{2}$ and $W_{3}$ denote the evaluation costs of the structured hashes $H_{2}$ and $H_{3}$, respectively. For an underlying hash output of Hamming weight *t*, direct evaluation requires one general hash computation and *t* group additions. Thus,

$$W_{2},W_{3}\approx T_{gh}+tT_{add}.$$

Using the benchmark timings $T_{gh}=0.006$ ms and $T_{add}=0.056$ ms, setting ℓ=256, and assuming an average Hamming weight of 128, we obtain

$$W_{2}=W_{3}\approx 7.174\;\mathrm{ms}.$$

This value is a model-based estimate under the stated assumptions, not a measured structured-hash runtime reported in the reference.

The same primitive-operation timings are applied to the operation counts of all compared schemes to provide a common cost model. This normalization does not imply that the schemes use identical cryptographic parameters or provide equivalent security levels. The choices ℓ=256 and average Hamming weight 128 are assumptions used to estimate the costs of $H_{2}$ and $H_{3}$ in the proposed scheme; they are not a security-level normalization for the other schemes.

Table 3 lists the operation symbols and benchmark timings. The comparison results for the computational costs of the schemes are presented in Table 4 and Table 5. These estimates cover the operations listed in Table 3. Unless included in $W_{2}$ and $W_{3}$, general hashing, group additions, scalar additions, and protocol-level checks are omitted. Accordingly, the reported values are operation-based estimates rather than complete execution-time measurements.

The adopted timings provide a common historical cost model for weighting primitive-operation counts. They do not establish equivalent contemporary security levels across the compared schemes. The resulting numerical comparisons therefore describe costs within this model rather than a definitive performance advantage at a common security level. No timing measurements from an implementation of the proposed scheme on UAV or representative embedded hardware are reported in this study.

Table 4 compares the computational costs of the schemes, where *n* denotes the number of proxy signers. Using the adopted benchmark timings, we estimate the costs of Dele-Generate, ProK-Generate, MP-Sign, and MP-Verify. The total cost is the sum of these four phases, as shown in Table 5. ProK-Generate includes delegation verification by all proxies, while MP-Sign includes partial signature generation, verification where specified, and aggregation.

For the scheme of Li et al. [19], the Dele-Generate, ProK-Generate, MP-Sign, and MP-Verify costs are 40.215 ms, 194.362n ms, 187.590n+33.582 ms, and 46.987n+133.970 ms, respectively. The total cost is 428.939n+207.767 ms.

For the scheme of Cao et al. [21], the corresponding costs are 60.392 ms, 178.708n ms, 259.788n+67.164 ms, and 33.582n+271.813 ms, giving a total of 472.078n+399.369 ms.

For the scheme of Jin et al. [23], the costs are 120.784 ms, 245.003n ms, 298.623n+177.839 ms, and 46.987n+245.003 ms. The total cost is 590.613n+543.626 ms.

For the scheme of Gu et al. [24], the costs are 53.620n ms, 173.316n ms, 40.215n ms, and 13.405n+238.742 ms, resulting in a total of 280.556n+238.742 ms. Its MP-Sign cost excludes individual partial signature verification before aggregation, which is not included in the construction.

For the scheme of Chen et al. [25], the costs are 60.392 ms, 165.303n ms, 218.923n+33.582 ms, and 33.582n+205.518 ms. The total cost is 417.808n+299.492 ms. Since this scheme has no separate partial proxy signing key generation procedure, the ProK-Generate entry accounts only for delegation verification.

For the scheme of Shin et al. [26], the costs are 60.392 ms, 192.113n ms, 224.826n+67.164 ms, and 33.582n+258.408 ms, giving a total of 450.521n+385.964 ms.

For the proposed IBMPS scheme, the costs of Dele-Generate, ProK-Generate, MP-Sign, and MP-Verify are estimated as 47.389 ms, 158.933n ms, 178.241n+7.174 ms, and 185.415 ms, respectively. The total estimated cost is 337.174n+239.978 ms.

Under the stated counting rules and hash-cost assumptions, our scheme has a lower estimated total cost than the schemes of Li, Cao, Jin, Chen, and Shin. Gu’s scheme has a lower total cost, but omits partial signature verification before aggregation. Our scheme combines this verification capability with a fixed number of pairings and scalar multiplications in final signature verification.

**Figure 4:**
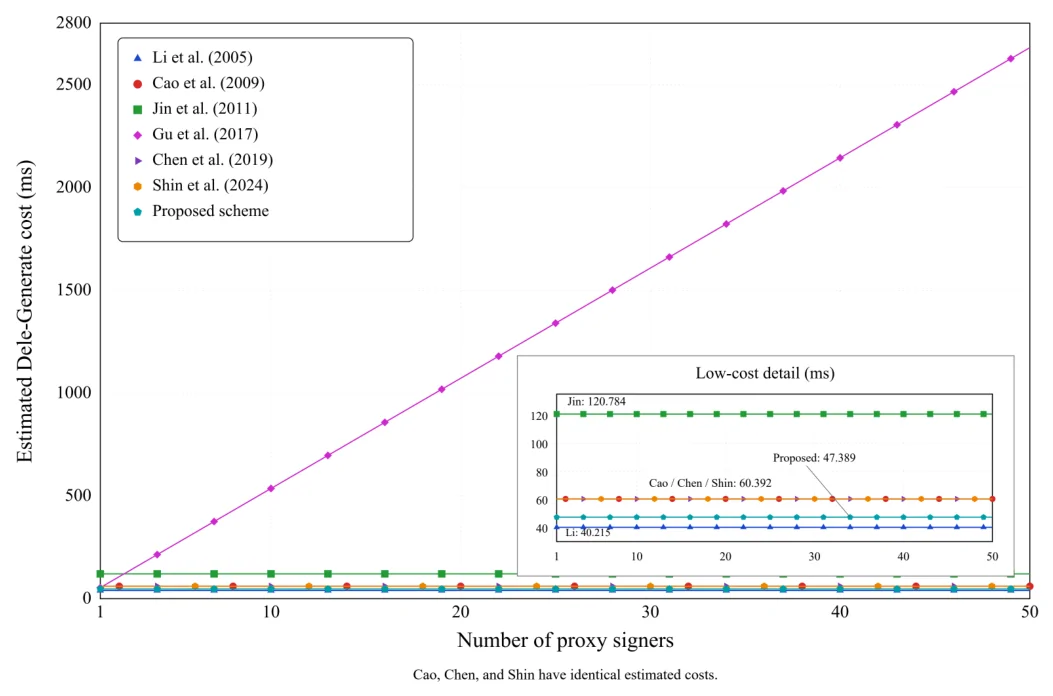
Comparison of the estimated computational costs of Delegate-Generate among the schemes of Li et al. [19], Cao et al. [21], Jin et al. [23], Gu et al. [24], Chen et al. [25], Shin et al. [26], and the proposed scheme.

As shown in Figure 4, the estimated Dele-Generate cost is independent of the number of proxy signers for all compared schemes except that of Gu et al. [24]. Li et al.’s scheme [19] has the lowest estimated cost of 40.215 ms, while our scheme requires 47.389 ms. The additional 7.174 ms comes from evaluating the structured hash $H_{2}$, corresponding to a 17.84% increase over Li et al.’s scheme. The cost of Gu et al.’s scheme is 53.620n ms and exceeds those of all other compared schemes when n≥3.

**Figure 5:**
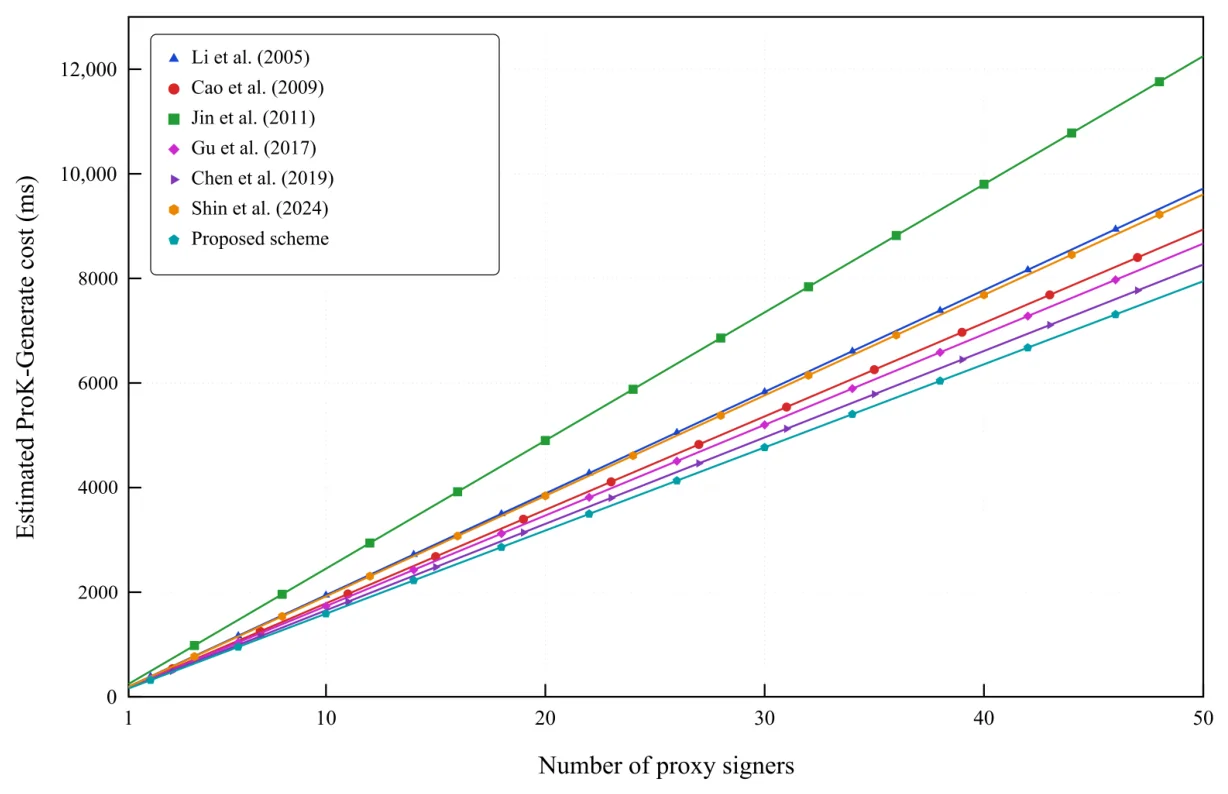
Comparison of the estimated computational costs of ProK-Generate among the schemes of Li et al. [19], Cao et al. [21], Jin et al. [23], Gu et al. [24], Chen et al. [25], Shin et al. [26], and the proposed scheme.

As shown in Figure 5, the estimated cost of the phase reported as ProK-Generate grows linearly with the number of proxy signers. This phase includes delegation verification and, where applicable, proxy signing-key generation. Chen et al.’s scheme [25] has no separate proxy signing-key generation procedure, so its entry accounts only for delegation verification. Under the adopted counting rules, our scheme has the lowest estimated cost of 158.933n ms, followed by Chen et al.’s scheme at 165.303n ms. Jin and Wen’s scheme [23] has the highest estimated cost of 245.003n ms, followed by Li et al.’s scheme [19] at 194.362n ms.

**Figure 6:**
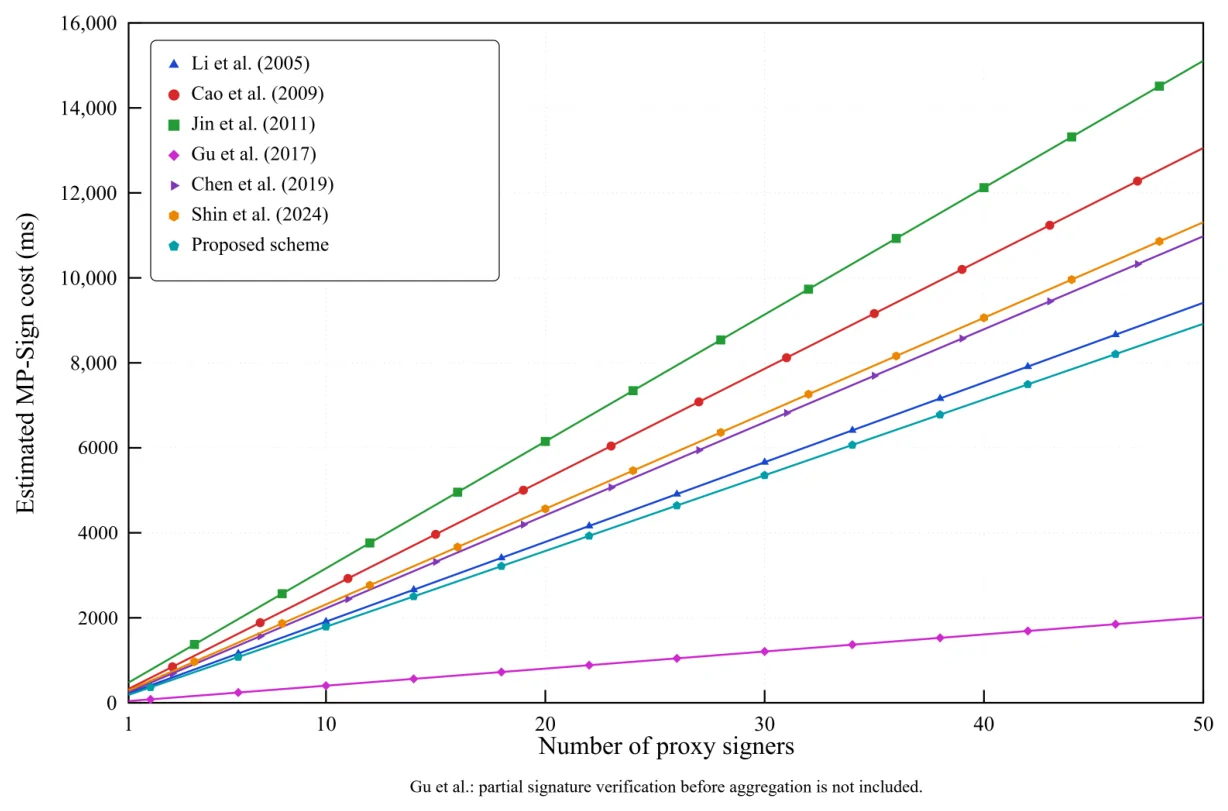
Comparison of the estimated computational costs of MP-Sign among the schemes of Li et al. [19], Cao et al. [21], Jin et al. [23], Gu et al. [24], Chen et al. [25], Shin et al. [26], and the proposed scheme.

As shown in Figure 6, the estimated MP-Sign costs grow linearly with the number of proxy signers. Jin and Wen’s scheme [23] has the highest estimated cost, followed by Cao et al.’s scheme [21]. Gu et al.’s scheme [24] has the lowest cost, but does not include partial signature verification before aggregation. Among the compared schemes that support this verification, our scheme has the lowest estimated MP-Sign cost under the adopted counting rules. Its cost is 178.241n+7.174 ms, compared with 187.590n+33.582 ms for Li et al.’s scheme [19]. The comparison with Gu et al.’s scheme therefore involves a trade-off between computational cost and partial signature verification.

**Figure 7:**
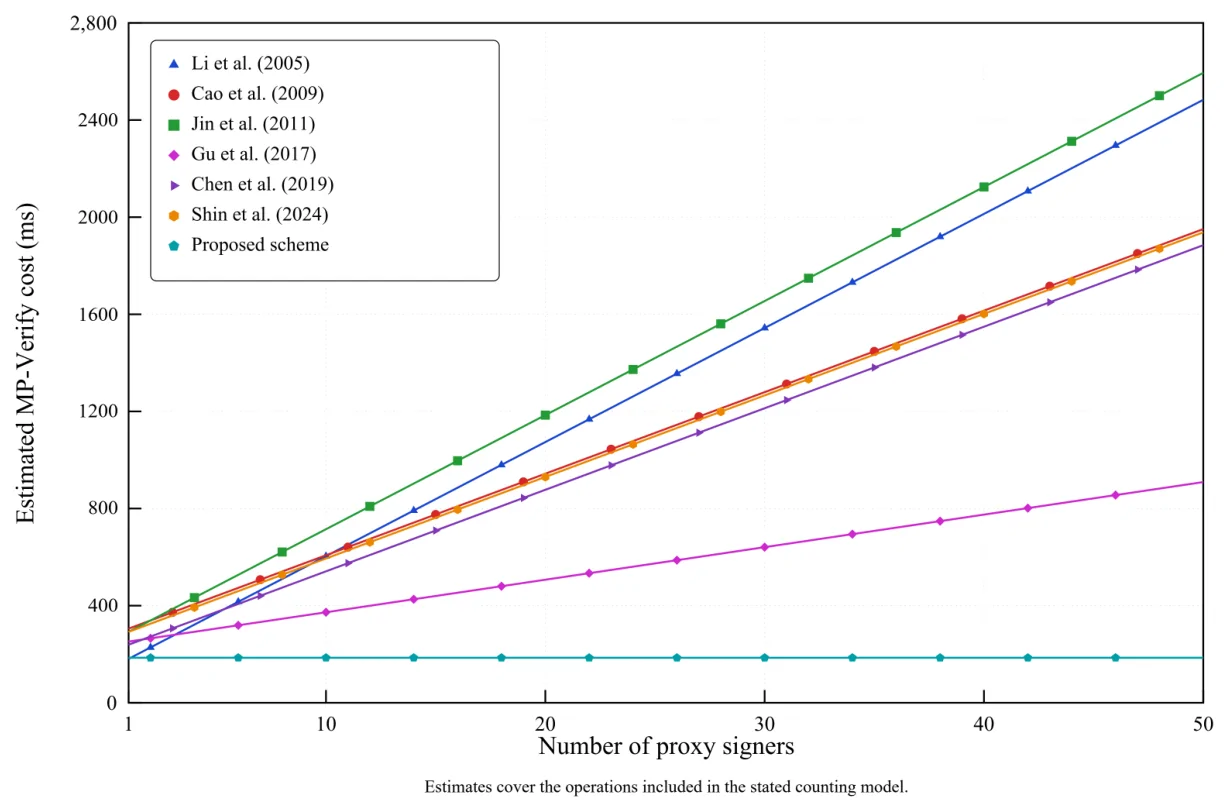
Comparison of the estimated computational costs of MP-Verify among the schemes of Li et al. [19], Cao et al. [21], Jin et al. [23], Gu et al. [24], Chen et al. [25], Shin et al. [26], and the proposed scheme.

As shown in Figure 7, the estimated MP-Verify costs of the other compared schemes increase linearly with the number of proxy signers. Under the adopted operation-count model, our scheme requires $3T_{sm}+4T_{bp}+W_{2}+W_{3}$, giving an estimated cost of 185.415 ms. At n=1, this is 4.458 ms higher than the cost of Li et al.’s scheme [19]; for n≥2, it is lower than the estimated costs of all six reference schemes. The fixed estimate concerns the operations included in the model. Processing signer identities, public-key lists, and warrant information still depends on the input size.

**Figure 8:**
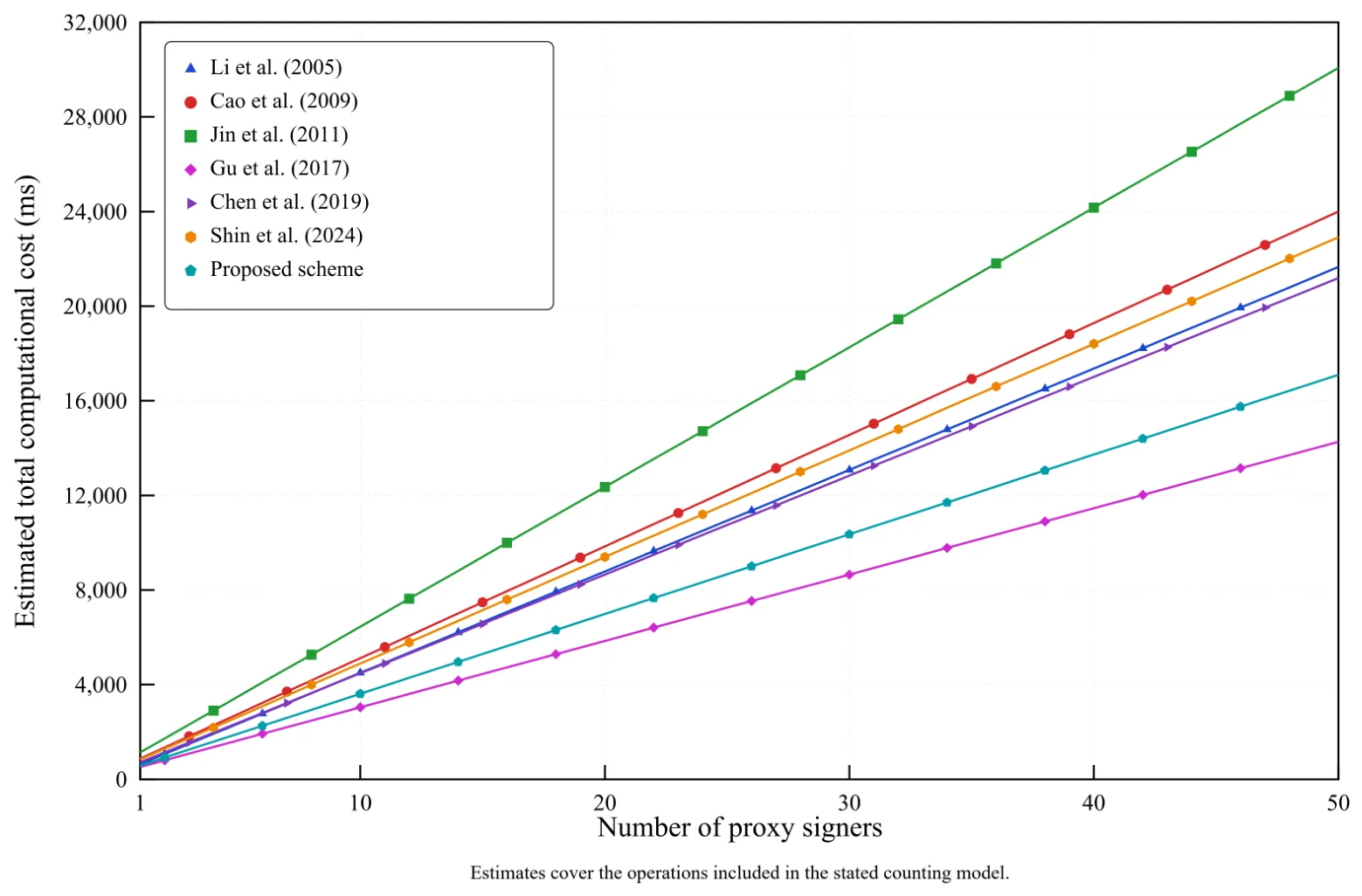
Comparison of the estimated total computational costs among the schemes of Li et al. [19], Cao et al. [21], Jin et al. [23], Gu et al. [24], Chen et al. [25], Shin et al. [26], and the proposed scheme.

As shown in Figure 8, the estimated total computational costs of all compared schemes increase linearly with the number of proxy signers *n*. Jin et al.’s scheme [23] has the highest estimated total cost, followed by Cao et al.’s scheme [21]. Gu et al.’s scheme [24] has the lowest cost, while the proposed scheme has the second-lowest cost under the adopted operation counts and benchmark assumptions. Although at n=10 this cost is 18.64% higher than that of Gu et al.’s scheme, our scheme additionally supports partial signature verification before aggregation. The comparison therefore reflects a trade-off between total computational cost and verification functionality. These estimates represent the aggregate computational cost across the four evaluated phases, rather than the end-to-end latency of a UAV rescue application.

For n=10, under the stated operation counts and structured-hash cost assumptions, the estimated Dele-Generate cost of our scheme is 47.389 ms. This is lower than those of Cao, Jin, Gu, Chen, and Shin by 21.53%, 60.77%, 91.16%, 21.53%, and 21.53%, respectively. Although it is 17.84% higher than Li’s cost, the absolute difference is only 7.174 ms and does not offset the savings in subsequent phases.

In ProK-Generate, our estimated cost is 1589.330 ms, which is lower than the corresponding costs of all six reference schemes. Compared with Li, Cao, Jin, Gu, Chen, and Shin, the reductions are 18.23%, 11.07%, 35.13%, 8.30%, 3.85%, and 17.27%, respectively. Within the adopted cost model, the preparation phase has a lower estimated cost than the corresponding entries of the six reference schemes, subject to their differences in functionality.

In MP-Sign, our estimated cost is 1789.584 ms, representing reductions of 6.28%, 32.85%, 43.44%, 19.49%, and 22.71% compared with Li, Cao, Jin, Chen, and Shin, respectively. Gu’s signing cost is lower, but its signing procedure does not include partial signature verification before aggregation. Our scheme checks each partial signature and rejects invalid inputs, enabling the coordinating UAV to detect invalid partial signatures before aggregation. The comparison with Gu must therefore account for both computational cost and verification functionality.

In MP-Verify, our estimated cost is 185.415 ms, which is lower than the corresponding costs of all six reference schemes. Compared with Li, Cao, Jin, Gu, Chen, and Shin, the reductions are 69.29%, 69.49%, 74.06%, 50.26%, 65.75%, and 68.80%, respectively. This estimate suggests potential savings in final verification under the adopted cost model. The actual computational burden at the ground control center remains to be measured.

Across the four phases, our total estimated cost is 3611.718 ms, which is lower than those of Li, Cao, Jin, Chen, and Shin by 19.69%, 29.46%, 44.00%, 19.34%, and 26.16%, respectively. Although the total is 18.64% higher than Gu’s, our scheme supports partial signature verification before aggregation and reduces the final verification cost by 50.26%. Under the adopted operation counts and historical timings, the scheme combines partial signature verification with lower estimated total costs than five reference schemes at n=10. These comparisons remain model-dependent and do not establish practical performance superiority on UAV hardware.

These results compare the encoded sizes of individual cryptographic objects rather than the complete end-to-end communication cost. The latter also includes distribution of the ordered public-key list *L*, exchange of proxy-key public values and partial signatures, and additional protocol messages or retransmissions.

Let $|G_{1}|$, $|G_{2}|$, and $|Z_{q}|$ denote the encoded lengths of a group element or scalar, rather than the cardinalities of the corresponding sets. Under the adopted representation, we assume $|G_{1}\left|=\right|G_{2}|=128$ bytes and $|Z_{q}|=20$ bytes. The warrant is denoted by *w*, with an assumed length of |w|=20 bytes. Table 6 compares the sizes of a user’s complete private key, a delegation delivered to one proxy, and a final multi-proxy signature. These sizes exclude public parameters, separately transmitted public keys, and protocol headers.

**Figure 9:**
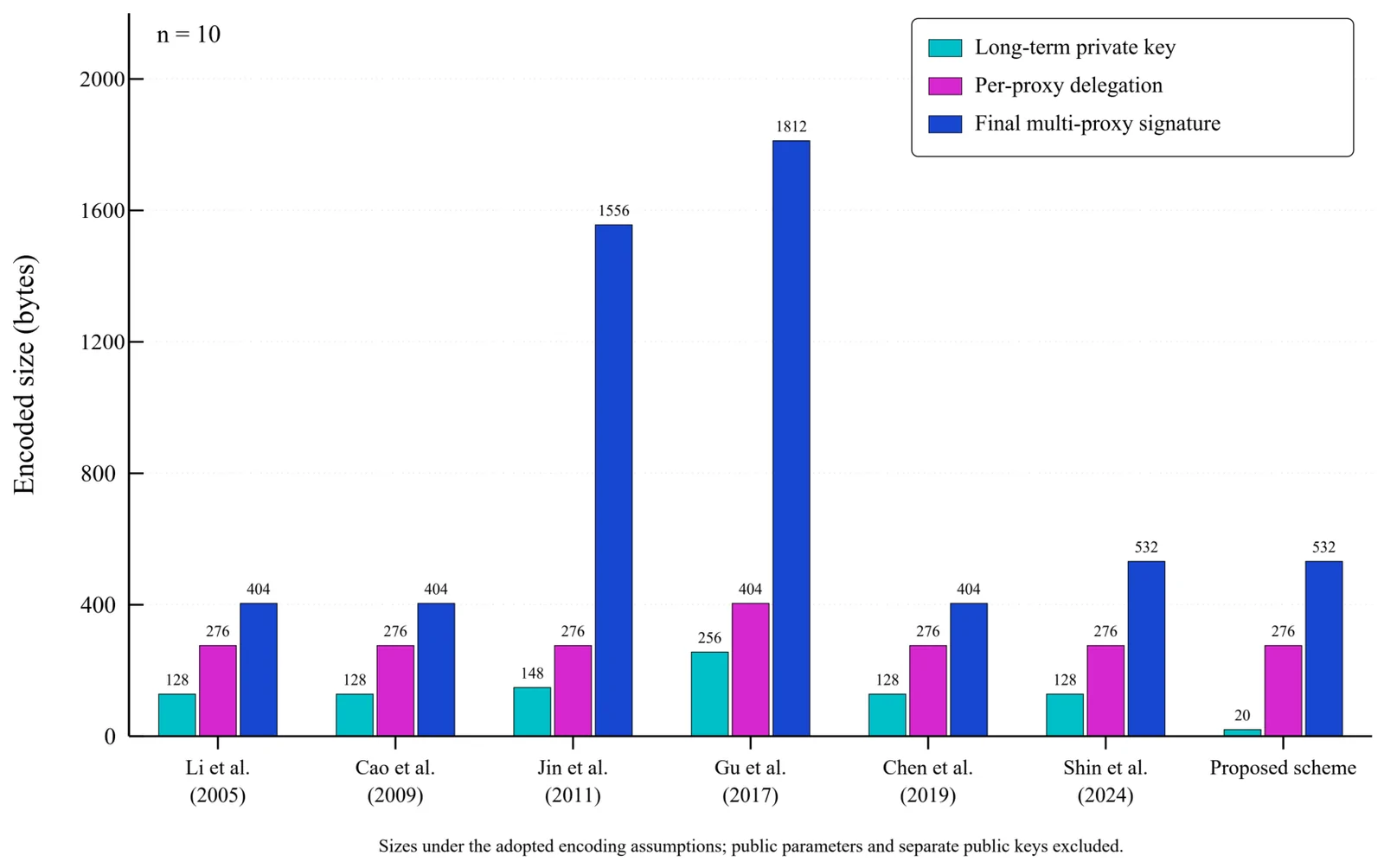
Comparison of private-key, delegation, and multi-proxy signature sizes among the schemes of Li et al. [19], Cao et al. [21], Jin et al. [23], Gu et al. [24], Chen et al. [25], Shin et al. [26], and the proposed scheme.

Under the adopted encoding assumptions, Figure 9 shows that the proposed scheme has the smallest long-term private-key size and matches the smallest per-proxy delegation size among the compared schemes. Its final multi-proxy signature has the same size as that of Shin et al.’s scheme [26], but is larger than those of the schemes of Li, Cao, and Chen [19,21,25]. The comparisons are summarized as follows:

- Private-key size: Our long-term private key occupies 20 bytes, corresponding to 15.63% of the 128-byte keys in [19,21,25,26], 13.51% of the 148-byte key in [23], and 7.81% of the 256-byte key in [24].
- Delegation size: Our per-proxy delegation occupies 276 bytes, matching the delegation sizes of the schemes in [19,21,23,25,26]. It is 68.32% of the 404-byte per-proxy delegation in [24].
- Multi-proxy signature size: Our final signature occupies 532 bytes, matching that of the scheme in [26]. It is 31.68% larger than the 404-byte signatures in [19,21,25]. Relative to the signature sizes of the schemes in [23,24], the corresponding ratios are
  $$\frac{532}{128n+276}\times 100\%\;\mathrm{and}\;\frac{532}{128n+532}\times 100\%,$$
  respectively. For n=10, these ratios are 34.19% and 29.36%.

## 8. Conclusions

This paper presented an identity-based multi-proxy signature scheme for UAV-assisted rescue operations. The construction integrates mission-specific delegation, individual proxy signing keys, and partial signature verification to authenticate the authorized UAV group’s joint endorsement of rescue reports. We formulated Game 1 to capture adaptive queries and signer collusion without public-key replacement, and proved unforgeability for its three forgery scenarios under the CDH assumption and collision-resistant hashing, with explicit reduction bounds. We also separately analyzed resistance to long-term private-key recovery under proxy signing-key exposure.

Under the adopted operation counts and historical benchmark timings, the cost model estimates lower preparation-phase and final-verification costs for the proposed scheme than for the six reference schemes. For n=10, it estimates a total cost 19.34–44.00% below that of five reference schemes, while Gu et al.’s scheme has a lower estimated total cost. These figures are model-based comparisons and do not establish an advantage at equivalent contemporary security levels or predict performance on UAV hardware. The proposed scheme also supports individual partial signature verification before aggregation, a functionality not provided by Gu et al.’s scheme. Compact long-term private-key storage and a fixed number of group elements in the final signature further support its potential applicability to collaborative UAV reporting. Future work will evaluate the scheme on UAV hardware and investigate its operation under intermittent connectivity and signer unavailability.

## Figures and Tables

**Table 1:** Notation and descriptions.

Notation	Description
params	Public system parameters.
mk	Master secret key.
$P_{pub}$	Master public key.
OS	Original signer.
${\left\{PS_{i}\right\}}_{i=1}^{n}$	Group of authorized proxy signers.
$PS_{i}$	The *i*-th proxy signer.
$PS_{j}$	Proxy signer designated as the clerk.
$ID_{OS}$	Identity of the original signer.
$ID_{PS_{i}}$	Identity of the *i*-th proxy signer.
${\left\{ID_{PS_{i}}\right\}}_{i=1}^{n}$	Set of identities of ${\left\{PS_{i}\right\}}_{i=1}^{n}$.
$d_{OS}/d_{PS_{i}}$	Private key of OS/$PS_{i}$.
$PK_{OS}/PK_{PS_{i}}$	Public key of OS/$PS_{i}$.
$PSK_{PS_{i}}$	Partial proxy signing key of $PS_{i}$.
*L*	Ordered public-key list of the designated proxy signers.
*w*	Warrant.
$\left(w,U_{OS},V_{OS}\right)$	Delegation.
*m*	Report (message).
$\sigma_{PS_{i}}$	Partial proxy signature generated by $PS_{i}$ on *m*.
$\sigma_{MPS}$	Multi-proxy signature on *m*.

**Table 2:** Security-related features and reported analyses of representative IBMPS schemes.

Scheme	PSK Generation	Reported Proof	Proof Model	Main Assumptions	Key-Recovery Resistance	PMPV
Xu [20]	Yes	No	N/A	N/A	N/A	No
Li [19]	Yes	No	N/A	N/A	No	Yes
Cao [21]	Yes	Yes	ROM	CDH	No	Yes
Gu [24]	Yes	Yes	SM	CDH	Yes	No
Chen [25]	No	Yes	ROM	CDH	N/A	Yes
Shin [26]	Yes	Yes	ROM	CDH	Yes	Yes
Proposed scheme	Yes	Yes	SM	CDH; CR for ${\hat{H}}_{2},{\hat{H}}_{3}$	Yes	Yes

PSK: Partial proxy signing key. PMPV: verification of individual partial multi-proxy signatures before aggregation. ROM: random oracle model. SM: standard model. CDH: computational Diffie–Hellman assumption. CR: collision resistance of ${\hat{H}}_{2}$ and ${\hat{H}}_{3}$. N/A: not applicable to the considered recovery mechanism or proof classification. Note: Key-recovery resistance refers to resistance to recovering the long-term private key following proxy signing-key (PSK) exposure under the conditions specified in the text. For the proposed scheme, the analysis assumes an honestly generated target public key that has not been replaced. This property does not prevent an adversary from signing with a compromised PSK.

**Table 3:** Operation symbols, benchmark timings, and structured-hash cost estimates.

Symbols	Definition
$T_{bp}$	Execution time of a bilinear pairing operation, $T_{bp}\approx$ 32.713 ms
$T_{htp}$	Execution time of a hash-to-point operation, $T_{htp}\approx$ 33.582 ms
$T_{sm}$	Execution time of a scalar multiplication operation in $G_{1}$, $T_{sm}\approx$ 13.405 ms
$T_{exp}$	Execution time of one exponentiation operation in $G_{2}$, $T_{exp}\approx$ 2.249 ms
$T_{gh}$	Benchmark execution time of one general hash computation: $T_{gh}\approx 0.006$ ms
$T_{add}$	Benchmark execution time of one group addition in $G_{1}$: $T_{add}\approx 0.056$ ms
$W_{2}$,$W_{3}$	Estimated evaluation times of the structured hash functions $H_{2}$ and $H_{3}$, respectively; $W_{2}=W_{3}\approx 7.174$ ms

*Note:* The timings are adopted from a historical benchmark. $W_{2}$ and $W_{3}$ are model-based estimates rather than measured runtimes of the structured hashes.

**Table 4:** Comparison of computational costs of various schemes.

Scheme	Dele-Generate	ProK-Generate	MP-Sign	MP-Verify
Li [19]	$3T_{sm}$	$2nT_{sm}+nT_{exp}+3nT_{bp}+2nT_{htp}$	$4nT_{sm}+nT_{exp}+3nT_{bp}+\left(n+1\right)T_{htp}$	$nT_{sm}+T_{exp}+3T_{bp}+\left(n+1\right)T_{htp}$
Cao [21]	$2T_{sm}+T_{htp}$	$nT_{sm}+3nT_{bp}+2nT_{htp}$	$2nT_{sm}+nT_{exp}+5nT_{bp}+\left(2n+2\right)T_{htp}$	$3T_{sm}+4T_{bp}+\left(n+3\right)T_{htp}$
Jin [23]	$4T_{sm}+2T_{htp}$	$nT_{sm}+4nT_{bp}+3nT_{htp}$	$\left(5n+1\right)T_{sm}+4\left(n+1\right)T_{bp}+\left(3n+1\right)T_{htp}$	$\left(n+1\right)T_{sm}+4T_{bp}+\left(n+3\right)T_{htp}$
Gu [24]	$4nT_{sm}$	$3nT_{sm}+nT_{exp}+4nT_{bp}$	$3nT_{sm}$	$\left(n+3\right)T_{sm}+T_{exp}+6T_{bp}$
Chen [25]	$2T_{sm}+T_{htp}$	$3nT_{bp}+2nT_{htp}$	$4nT_{sm}+3nT_{bp}+\left(2n+1\right)T_{htp}$	$3T_{sm}+3T_{bp}+\left(n+2\right)T_{htp}$
Shin [26]	$2T_{sm}+T_{htp}$	$2nT_{sm}+3nT_{bp}+2nT_{htp}$	$2nT_{sm}+4nT_{bp}+\left(2n+2\right)T_{htp}$	$2T_{sm}+4T_{bp}+\left(n+3\right)T_{htp}$
Proposed scheme	$3T_{sm}+W_{2}$	$4nT_{sm}+3nT_{bp}+nW_{2}$	$3nT_{sm}+4nT_{bp}+W_{2}+nW_{3}$	$3T_{sm}+4T_{bp}+W_{2}+W_{3}$

**Table 5:** Comparison of total computational costs.

Scheme	Total Cost Expression	Estimated Total Cost (ms)
Li [19]	$\begin{array}{cc} & \left(7n+3\right)T_{sm}+\left(2n+1\right)T_{exp}\\ & +\left(6n+3\right)T_{bp}+\left(4n+2\right)T_{htp} \end{array}$	428.939n+207.767
Cao [21]	$\begin{array}{cc} & \left(3n+5\right)T_{sm}+nT_{exp}\\ & +\left(8n+4\right)T_{bp}+\left(5n+6\right)T_{htp} \end{array}$	472.078n+399.369
Jin [23]	$\begin{array}{cc} & \left(7n+6\right)T_{sm}+\left(8n+8\right)T_{bp}\\ & +\left(7n+6\right)T_{htp} \end{array}$	590.613n+543.626
Gu [24]	$\begin{array}{cc} & \left(11n+3\right)T_{sm}+\left(n+1\right)T_{exp}\\ & +\left(4n+6\right)T_{bp} \end{array}$	280.556n+238.742
Chen [25]	$\begin{array}{cc} & \left(4n+5\right)T_{sm}+\left(6n+3\right)T_{bp}\\ & +\left(5n+4\right)T_{htp} \end{array}$	417.808n+299.492
Shin [26]	$\begin{array}{cc} & \left(4n+4\right)T_{sm}+\left(7n+4\right)T_{bp}\\ & +\left(5n+6\right)T_{htp} \end{array}$	450.521n+385.964
Proposed scheme	$\begin{array}{cc} & \left(7n+6\right)T_{sm}+\left(7n+4\right)T_{bp}\\ & +\left(n+3\right)W_{2}+\left(n+1\right)W_{3} \end{array}$	337.174n+239.978

*Note:* The numerical totals are analytical estimates under the adopted operation counts and historical timings. Equivalent contemporary security levels have not been verified across the schemes.

**Table 6:** Comparison of private-key, delegation, and multi-proxy signature sizes.

Scheme	Private Key (Bytes)	Delegation (Bytes)	Multi-Proxy Signature (Bytes)
Li [19]	$|G_{1}|=128$	$\left|w|+2\right|G_{1}|=276$	$\left|w|+3\right|G_{1}|=404$
Cao [21]	$|G_{1}|=128$	$\left|w|+2\right|G_{1}|=276$	$\left|w|+3\right|G_{1}|=404$
Jin [23]	$|G_{1}\left|+\right|Z_{q}|=148$	$\left|w|+2\right|G_{1}|=276$	$\left|w|+\left(n+2\right)\right|G_{1}|=128n+276$
Gu [24]	$2|G_{1}|=256$	$\left|w|+3\right|G_{1}|=404$	$\left|w|+\left(n+4\right)\right|G_{1}|=128n+532$
Chen [25]	$|G_{1}|=128$	$\left|w|+2\right|G_{1}|=276$	$\left|w|+3\right|G_{1}|=404$
Shin [26]	$|G_{1}|=128$	$\left|w|+2\right|G_{1}|=276$	$\left|w|+4\right|G_{1}|=532$
Proposed scheme	$|Z_{q}|=20$	$\left|w|+2\right|G_{1}|=276$	$\left|w|+4\right|G_{1}|=532$

*Note:* The reported sizes describe individual object representations and exclude the complete protocol communication overhead.

## Data Availability

The original contributions presented in this study are included in the article. Further inquiries can be directed to the corresponding author.
